# Comparative Genomics Analysis of *Vibrio anguillarum* Isolated from Lumpfish (*Cyclopterus lumpus*) in Newfoundland Reveal Novel Chromosomal Organizations

**DOI:** 10.3390/microorganisms8111666

**Published:** 2020-10-27

**Authors:** Ignacio Vasquez, Trung Cao, Setu Chakraborty, Hajarooba Gnanagobal, Nicole O’Brien, Jennifer Monk, Danny Boyce, Jillian D. Westcott, Javier Santander

**Affiliations:** 1 Microbial Pathogenesis and Vaccinology Laboratory, Department of Ocean Sciences, Memorial University, Logy Bay, NL A1C 5S7, Canada; ivasquezsoli@mun.ca (I.V.); ttcao@mun.ca (T.C.); schakraborty@mun.ca (S.C.); hgnanagobal@mun.ca (H.G.); 2 Department of Fisheries and Land Resources, Aquatic Animal Health Division, Government of Newfoundland and Labrador, St. John’s, NL A1E 3Y5, Canada; nicoleobrien@gov.nl.ca; 3 Dr. Joe Brown Aquatic Research Building (JBARB), Department of Ocean Sciences, Memorial University of Newfoundland, Logy Bay, NL A1C 5S7, Canada; jmonk@mun.ca (J.M.); dboyce@mun.ca (D.B.); 4 Fisheries and Marine Institute, Memorial University of Newfoundland, St. John’s, NL A1C 5R3, Canada; Jillian.Westcott@mi.mun.ca

**Keywords:** *Vibrio anguillarum*, lumpfish, genomics, insertion elements, evolution

## Abstract

*Vibrio anguillarum* is a Gram-negative marine pathogen causative agent of vibriosis in a wide range of hosts, including invertebrates and teleosts. Lumpfish (*Cyclopterus lumpus*), a native fish of the North Atlantic Ocean, is utilized as cleaner fish to control sea lice (*Lepeophtheirus salmonis*) infestations in the Atlantic salmon (*Salmo salar*) aquaculture industry. *V. anguillarum* is one of the most frequent bacterial pathogens affecting lumpfish. Here, we described the phenotype and genomic characteristics of *V. anguillarum* strain J360 isolated from infected cultured lumpfish in Newfoundland, Canada. Koch’s postulates determined in naïve lumpfish showed lethal acute vibriosis in lumpfish. The *V. anguillarum* J360 genome was shown to be composed of two chromosomes and two plasmids with a total genome size of 4.56 Mb with 44.85% G + C content. Phylogenetic and comparative analyses showed that *V. anguillarum* J360 is closely related to *V. anguillarum* strain VIB43, isolated in Scotland, with a 99.8% genome identity. Differences in the genomic organization were identified and associated with insertion sequence elements (ISs). Additionally, *V. anguillarum* J360 does not possess a pJM1-like plasmid, typically present in virulent isolates from the Pacific Ocean, suggesting that acquisition of this extrachromosomal element and the virulence of *V. anguillarum* J360 or other Atlantic isolates could increase.

## 1. Introduction 

*Vibrio* spp. is naturally ubiquitous in aquatic and marine environments [1]. Some members of this genus can cause infections in humans after exposition to contaminated water, such as *Vibrio cholerae,* the causative agent of cholera, and after consumption of raw contaminated seafood, such as *V. parahaemolitycus*, *V. alginolyticus*, and *V. vulnificus* [1,2,3]. Other members of *Vibrio* spp. such as *V. splendidus*, *V. nereis*, *V. harveyi*, *V. damsela*, *V. tubiashi*, and *V. anguillarum* are pathogens of aquatic organisms including cultured fish species [4,5]. *V. anguillarum* is a Gram-negative marine pathogen, which causes vibriosis in a wide range of cultured and wild invertebrates and teleosts hosts, but is also present in brackish and fresh water [6,7,8].

The lumpfish (*Cyclopterus lumpus*), a native fish of the North Atlantic Ocean, is utilized as a cleaner fish to control sea lice (*Lepeophtheirus salmonis*) infestations in the Atlantic salmon (*Salmo salar*) industry [9,10,11]. The lumpfish performs well in cold environments, removing nearly 90% of the sea lice at sea-cages (with a feeding rate of 0.3 sea-lice per day) [9,12,13]. Lumpfish have been reported to be up to 64% more efficacious with respect to sea lice removal when compared to other wrasse species utilized as cleaner fish species, like ballan (*Labrus bergylta*), corkwing (*Crenilabrus melops*), rock cook (*Centrolabrus exoletus*), and goldsinny (*Ctenolabrus rupestris*) [10,12]. In addition, these wrasses (not including lumpfish) exhibit a reduced activity during winter, because they enter into a hypometabolic state (similar to hibernation) [13,14], and this eventually decreases sea lice removal efficiency [15,16].

*V. anguillarum* is one of the most frequent pathogens affecting lumpfish in sea-cages [9,10]. In lumpfish, *V. anguillarum* causes hemorrhagic septicemia, which is characterized by skin lesions, gill hemorrhages, and bacterial aggregations in lymphoid organs [10,17,18]. In Atlantic Canada, *V. anguillarum* is frequent [19], but it has not been reported in lumpfish.

Currently, 23 serotypes of *V. anguillarum* have been described, and serotypes O1, O2, and O3 are the most frequent serotypes causing outbreaks in teleosts [18,19]. The *V. anguillarum* genome consists of two chromosomes, and a large virulent plasmid is present in some isolates [8]. Several virulent strains of *V. anguillarum* from different geographic locations and fish species have been sequenced [20], including *V. anguillarum* M3 [7], *V. anguillarum* NB10 [6], and *V. anguillarum* 775 [8]. *V. anguillarum* is more frequently reported in warm-water fish, and several of these genomes have been sequenced, assembled, and annotated, and although virulent strains of *V. anguillarum* have been isolated from cold water environments, only a few of these isolates have been described [20].

In this study, we describe the complete genome of a *V. anguillarum* strain isolated from an outbreak in cultured lumpfish in Newfoundland, Canada, and compared its genome to other known *V. anguillarum* strains. We determined that *V. anguillarum* J360 produces acute vibriosis in lumpfish, does not harbor virulent plasmids, is closely related to *V. anguillarum* strains isolated from the Atlantic coasts, and is distantly related to *V. anguillarum* strains isolated from the Pacific coasts. *V. anguillarum* J360 also showed high similarity to a *V. anguillarum* isolated from sea bass (*Dicentrarchus labrax*) in Scotland. Comparative analysis suggested that insertion sequence elements play a key role in *V. anguillarum* evolution.

## 2. Material and Methods

### 2.1. Bacterial Culture Conditions

A single colony of *V. anguillarum* J360 was grown routinely in 3 mL of Trypticase soy broth (TSB, Difco, Franklin Lakes, NJ, USA) supplemented with up to 2% NaCl at 15 °C in a 16 mm-diameter glass tube and placed in a roller for 24 h. When required, the culture medium was supplemented with 100 µM of FeCl_3_, 100 µM 2,2-dipyridyl, or 1.5% bacto-agar (Difco). Chrome Azurol S (CAS) plates were used for the siderophores secretion assay [21]. Blood agar plates (0.5% salmon blood) were used to evaluate hemolytic activity. Bacterial cells were harvested at mid-log phase, at an optical density (O.D. 600 nm) of ≈0.7 (≈4.1 × 10^8^ CFU/mL), and washed three times with phosphate-buffered saline (PBS; 136 mM NaCl, 2.7 mM KCl, 10.1 mM Na_2_HPO_4_, 1.5 mM KH_2_PO_4_ (pH 7.2)) [22] at 4200× *g* for 10 min at room temperature.

### 2.2. V. anguillarum Isolation

*V. anguillarum* strain J360 was isolated from the head kidney of infected cultured lumpfish in Newfoundland, Canada. Fish with classic vibriosis symptoms were netted and immediately euthanized with an overdose of MS222 (400 mg/L) (Syndel Laboratories, Nanaimo, BC, Canada). Tissue samples (e.g., head kidney, liver, and spleen) were collected and placed into sterile homogenized bags (Nasco whirl-pak^®^, Fort Atkinson, WI, USA). The infected tissues were weighed and homogenized in PBS up to a final volume of 1 mL. One hundred microliters of the homogenized tissue suspension were plated onto Trypticase soy agar (TSA) plates and incubated at 15 °C for 48 h. Isolated colonies were selected and purified for further analysis. Bacteria stock was preserved at −80 °C in 10% glycerol and 1% peptone solution.

### 2.3. Matrix-Assisted Laser Desorption/Ionization—Time-of-Flight (MALDI-TOF) Mass Spectrometry and Serotypification Analysis

Serotypification and MALDI-TOF were conducted commercially at the University of Prince Edward Island, Canada. The MALDI Biotyper RTC was conducted according to the MALDI Biotyper 3.1 user manual and parameter settings as previously published [23].

### 2.4. Biochemical, Enzymatic, and Physiological Characterization

*V. angillarum* J360 growth curves were conducted in triplicates at 15 °C, 28 °C, and under rich and iron-limited conditions according to established protocols [24]. Briefly, a single colony of *V. angillarum* J360 was inoculated in 3 mL of TSB and incubated in a roller for 24 h at 15 °C. An inoculum of 300 μL of cells at mid-log phase (OD_600_ ≈ 0.7) was added to 30 mL of fresh TSB into 250 mL flasks and incubated for 48 h at 15 °C and 28 °C with aeration (180 rpm) in an orbital shaker. Bacterial growth was monitored spectrophotometrically until OD_600_ ≈ 2.0 ± 0.3 (≈8 × 10^8^ CFU/mL) using a Genesys 10 UV spectrophotometer (Thermo Spectronic, Thermo Fischer Scientific, USA). Growth curves under iron-limited conditions were determined using three different 2,2-dipyridyl concentrations (100, 150, 250 µM). Controls consisted of nonsupplemented TSB. The doubling time was estimated using the optical density (OD) values, *g* = *b* − *B*, where *b* is the OD value at the end of the time interval and *B* is the OD value at the beginning of the time interval.

The biochemical profile of *V. anguillarum* J360 was characterized using API20E, API20NE, and APY ZYM systems (BioMerieux, Marcy-l’Etoile, France) according to the manufacturer’s instructions. The strips were incubated at 15 °C for 48 h and the results were analyzed using APIweb (BioMerieux). *V. anguillarum* J360 growth was also tested at different temperatures (4 °C, 15 °C, 28 °C, 37 °C) and different concentrations of NaCl (0%, 0.5%, 2%). Motility, hemolysin on TSA (5% salmon blood), siderophore synthesis, catalase activity, and oxidase activity were evaluated using standard methods [25]. The antibiogram of *V. anguillarum* J360 was determined for tetracycline (10 mg/mL), oxytetracycline (30 mg/mL), ampicillin (10 mg/mL), sulfamethoxazole (STX) (25 mg/mL), chloramphenicol (30 mg/mL), colistin sulphate (10 mg/mL), and oxalinic acid (2 mg/mL) using standard methods [25,26].

### 2.5. Siderophores Synthesis

*V. anguillarum* J360 was grown under conditions previously described. Bacterial cells were harvested at mid-log phase, at an optical density (OD_600_) of ≈0.7 (≈4.1 × 10^8^ CFU/mL), washed three times with phosphate-buffered saline (PBS; 136 mM NaCl, 2.7 mM KCl, 10.1 mM Na_2_HPO_4_, 1.5 mM KH_2_PO_4_ (pH 7.2)) [22] at 4200× *g* for 10 min at room temperature, and resuspended in 1 mL of PBS. An inoculum of 300 µL of the bacterial suspension was added to 3 mL TSB medium and TSB medium supplemented with 100 µM of FeCl_3_ or 100 µM of 2,2-dipyridyl. *V. anguillarum* J360 was grown at 15 °C for 24 h with aeriation. Following the incubation period, the cells were harvested at mid-log phase, washed twice with PBS, and resuspended in 100 µL of PBS. Five microliters of the concentrated bacterial pellet was inoculated onto CAS agar plates [21] and incubated at 15 °C and 28 °C for 48 h.

### 2.6. Fish Holding

Fish were produced and maintained at the Dr. Joe Brown Aquatic Research Building (JBARB), Memorial University of Newfoundland (MUN), under the animal protocols #18-1-JS and #18-03-JS. Lumpfish were acclimated to ≈8–10 °C in 500 L tanks supplied with 95–110% air-saturated and UV-treated filtered flow-through seawater, and an ambient photoperiod. Stocking density was maintained at 6.6 kg/m^3^. The fish were fed daily by automated feeders a commercial diet (Skretting—Europa; 15 crude protein (55%), crude fat (15%), crude fiber (1.5%), calcium (3%), phosphorus (2%), sodium (1%), vitamin A (5000 IU/kg), vitamin D (3000 IU/kg), and vitamin E (200 IU/kg))) at a rate of 0.5% of their body weight per day.

### 2.7. Infection Assay in Lumpfish

Naive lumpfish (weight ≈ 55 g) were transferred from the JBARB to the AQ3 biocontainment Cold-Ocean Deep-Sea Research Facility (CDRF) for infection assays. Fish were separated into three 500 L tanks containing 60 fish per dose and acclimated for 2 weeks under previously described conditions. The infection procedures were conducted according to established protocols [27]. Briefly, fish were anesthetized with 40 mg of MS222 (Syndel Laboratories, BC, Canada) per liter of sea water and intraperitoneally infected with 100 µL of 10^6^, or 10^7^ CFU per dose of *V. anguillarum* J360. The Control group (*n* = 60) was mock-injected with PBS. Mortality was monitored daily until 30 days post-infection. Samples of liver, spleen, and head kidney were taken from moribund fish to re-isolate the pathogen.

### 2.8. DNA Extraction and Sequencing

*V. anguillarum* J360 was grown under conditions previously described. Bacterial cells were harvested at mid-log phase, at an optical density OD_600_ of ≈0.7 (≈4.1 × 10^8^ CFU/mL) and washed three times with PBS. DNA extraction was conducted using a Wizard DNA extraction High Molecular Weight Kit (Promega, Madison, WI, USA). The DNA integrity and purity were evaluated by gel electrophoresis (agarose gel 0.8%) [28] and spectrophotometry (Genova-Nano Spectrophotometer, Jenway, UK). Libraries and sequencing were conducted commercially at Genome Quebec (Montreal, QC, Canada) using PacBio RS II and Miseq Illumina sequencers.

### 2.9. Genome Assembly, Annotation, and Mapping

PacBio reads were assembled at Genome Quebec using Celera Assembler (August 2013 version). Annotation was completed using the Rapid Annotation Subsystem Technology pipeline (RAST) (http://rast.nmpdr.org/) [29]. The two *V. anguillarum* J360 chromosomes and large plasmid were submitted to the National Center for Biotechnology Information (NCBI) and re-annotated using the NCBI Prokaryotic Genome Annotation Pipeline.

To detect small plasmids, the Illumina reads were trimmed using CLC Genomics Workbench v20.0 (CLC Bio) and examined for quality using FastQC version 12 [30]. High-quality Illumina reads were assembled using the CLC Genomics Workbench de novo tool and aligned to the reference *V. anguillarum* J360 chromosomes and large plasmid using the genome finishing module tools with default parameters. Illumina sequences that did not align with the chromosomes or large plasmid were analyzed and annotated using the previously described methods. The *V. anguillarum* J360 whole genome was mapped by using DNA plotter software [31].

### 2.10. Whole Genome Comparison and Phylogeny Analysis

The genomes utilized are listed in Table 1. Whole genomes were aligned to calculate the average nucleotide identity (ANI) using the CLC Genomic workbench v20 (CLC Bio) whole genome analysis tool with default parameters (Min. initial seed length = 15; Allow mismatches = yes; Min. alignment block = 100). A minimum similarity (0.8) and a minimum length (0.8) were used as parameters for CDS identity. A comparative heat map was constructed using the heat map tool with default parameters (Euclidean distance method and complete cluster linkages). Phylogenetic analysis was performed in two different software, CLC Genomic workbench v20.0 and MEGA X [32], with the same parameters for robustness comparison purposes, using the extracted alignment from the ANI analysis. Evolutionary history was calculated using the neighbor-joining method [33] with a bootstrap consensus of 500 replicates, and evolutionary distance was computed using the Jukes–Cantor method [34]. *Photobacterium damselae* 91–197 (AP018045/6) chromosomes were utilized as outgroups [35]. Whole genome dot plots between closely related *V. anguillarum* strains were constructed using the CLC Genomic workbench v20.0, a whole genome analysis tool to visualize and further analyze genomic differences. Comparative alignment analysis represents homologous regions, translocations, and inversions within the two strain genomes for chromosome-I and for chromosome-II. Homologous regions were identified as locally colinear blocks (LCBs), which represent conserved regions that do not present rearrangements, and genomic gaps (GGs) were identified as unmatched regions. Analysis was performed using the progressive Mauve v20150206 [36].

### 2.11. Multi-Locus Sequence Analysis of V. anguillarum Housekeeping Genes

Multi-locus analysis (MLSA) was used to infer the phylogeny history of *V. anguillarum* strains using reference genes including 16S ribosomal RNA subunit (*rrn*), cell-division protein (*ftsZ*), glyceraldehyde-3-phosphate dehydrogenase (*gapA*), gyrase beta subunit (*gyrB*), rod shape-determining protein (*mreB*), uridine monophosphate (UMP) kinase or uridylate kinase (*pyrH*), recombinase A (*recA*), RNA polymerase alpha subunit (*rpoA*), and topoisomerase I (*topA*) gene sequences. Only genes from complete genomes were considered for the MLSA. Sequences were aligned using CLC Genomic workbench v20.0 (CLC Bio). Concatenation of locus sequences was made using Sequence Matrix software v1.7.8 [42]. Phylogenetic analysis was performed using the two software mentioned above with the same parameters. Evolutionary history was estimated using the neighbor-joining method [33] with a bootstrap consensus of 500 replicates, and evolutionary distance was computed using the Jukes–Cantor method [34]. The gene loci and accession numbers are listed in Table S1.

### 2.12. Genomic Islands Analysis

Detection of genomic islands (GIs) was conducted using IslandViewer v.4 pipeline (https://www.pathogenomics.sfu.ca/islandviewer/browse/), which integrate IslandPath-DIMOB, SIGH-HMM, and IslandPick analysis tools into a single analysis [43]. Analysis was performed for the chromosomes and the plasmids.

### 2.13. Comparative Analysis of V. aguillarum J360 Large Plasmid

A genomic comparison of virulent and nonvirulent plasmids between *V. anguillaurm* species was performed using the whole genome alignment tool of CLC Genomics workbench v20.0 with default parameters. Plasmids used in this analysis were: p292-VIB12 (CP023312); p15-VIB43 (CP023056); pVaM3 (CP006701); pJM1 (AY312585); p67vangNB10 (LK021128); and p65-ATCC 68554 (CP023210). Plasmids were aligned to calculate the ANI. A comparative heat map was constructed using the heat map tool with default parameters (Euclidean distance method and complete cluster linkages).

### 2.14. Statistical Analysis

Fish survival percentages were transformed to arc-sin ($\sqrt{survival}\;rate\;ratio$). One-way ANOVA was used to determine significance, followed by Tukey’s post-hoc test, to determine significant differences (*p* < 0.05). All statistical analyses were performed using GraphPad Prism 7 (GraphPad Software, California, CA, USA).

### 2.15. Ethics Statement

All animal protocols required for this research were approved by the Institutional Animal Care Committee and the Biosafety Committee at Memorial University of Newfoundland (MUN). Animal assays were conducted under protocols #16-92-KG, #18-01-JS #18-03-JS, and biohazard license L-01, approved on 26-05-2020.

## 3. Results

### 3.1. Phenotypic, Biochemical, and Enzymatic Characterization

**Figure 1:**
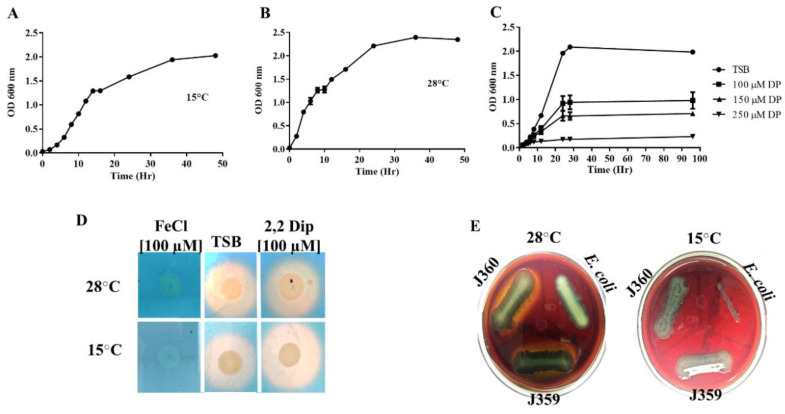
Bacterial growth and physiological characteristics of *V. anguillarum* J360. (**A**) *V. anguillarum* J360 growth in Trypticase soy broth (TSB) at 15 °C for 48 h. Independent triplicates were utilized. (**B**) *V. anguillarum* J360 growth in TSB at 28 °C for 48 h. Independent triplicates were utilized. (**C**) *V. anguillarum* J360 growth under iron-limited conditions (TSB supplemented with 100, 150, and 250 µM of 2,2-dipyridyl). (**E**) *V. anguillarum* J360 hemolysin activity assay on Sheep blood agar plates incubated at 15 °C and 28 °C for 48 h. (**D**) Siderophore synthesis on chrome azurol S (CAS) agar plates from *V. anguillarum* J360 grown under iron-enriched conditions (TSB supplemented with 100 µM of FeCl_3_), standard culture conditions (TSB), and iron-limited conditions (TSB supplemented with 100 µM of 2,2-dipyridyl). Yellow reaction around the *V. anguillarum* J360 colony indicated positive for siderophore synthesis.

*V. anguillarum* strain J360 was capable of growing in Tryptic soy broth (TSB) and Luria Bertani (LB) medium up to 30 °C (Table 2). The *V. anguillarum* J360 doubling time in TSB supplemented with 2% NaCl at 15 °C was 2 h (Figure 1A) and 1 h at 28 °C (Figure 1B). *V. anguillarum* J360 did not grow at 37 °C, in TCBS selective media at 15 and 28 °C, or in the absence of NaCl. *V. anguillarum* J360 was shown to be motile and capable of synthesizing type I fimbria, oxidase, and catalase (Table 2). The antibiogram analysis showed that *V. anguillarum* J360 is ampicillin-resistant and susceptible to tetracycline, oxytetracycline, sulfamethoxazole, chloramphenicol, colistin sulphate, oxalinic acid, and O-129 (Table 2).

*V. anguillarum* J360 growth under iron-limited conditions was evaluated at 15 °C on TSB media with different 2,2-dipyridyl concentrations (100, 150, 250 µM). *V. anguillarum* J360 was able to grow in the presence of high concentrations of 2,2-dipyridyl. The doubling time at 100 µM of 2,2-dipyridyl was 3 h (Figure 1C); however, it was increased to 4 h (OD_600_ ≈ 0.7) and 5 h (OD_600_ ≈ 0.2) at 150 and 250 µM of 2,2-dipyridyl, respectively (Figure 1C). No siderophores secretion was observed in the cells grown under iron-enriched conditions. Additionally, there was no significant differences in the size of the halo for siderophores secretion between nonsupplemented TSB and supplemented TSB with 100 µM of 2,2-dipyridyl at 28 °C (Figure 1D). Nonetheless, a small difference in the halo increased diameter was observed for siderophores secretion under iron-limited conditions at 15 °C (Figure 1D). Hemolytic activity was evaluated on blood agar plates at 28 °C and 15 °C. *V. anguillarum* J360 hemolytic activity was observed only at 28 °C (Figure 1E).

The biochemical and enzymatic profiles indicate that *V. anguillarum* J360 is able to synthetize alkaline phosphatase, esterase (C_4_), esterase lipase (C_8_), lipase (C_14_), leucine, valine and cysteine arylamidase, and acid phosphatase (Table S2). *V. anguillarum* J360 reduces nitrates and glucose; produces indole from tryptophan; produces urease, β-galactosidase, arginine hydrolase, esculinase and gelatinase; and is able to utilize arabinose, mannose, mannitol, N-acetyl-glucosamine, and maltose (Tables S3 and S4). The API20NE profile indicated that the isolate was *V. fluvialis,* with 99.7% probability (Table S3).

### 3.2. MALDI-TOF and Agglutination Analysis

The MALDI-TOF mass spectrometry score for *V. anguillarum* was 1.96, indicating that there was a low confidence for identification. The *V. anguillarum* agglutination test was positive for the O2 serotype and negative for the O1 serotype.

### 3.3. Infection Assay in Specific Pathogen-Free Lumpfish

**Figure 2:**
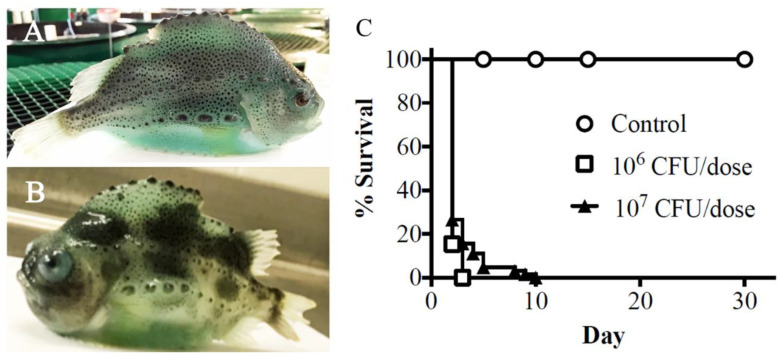
Pathogenicity and virulence of *V. anguillarum* J360 in lumpfish (*C. lumpus*). (**A**) Healthy lumpfish cultured at Dr. Joe Brown Aquatic Research Building (JBARB). (**B**) Lumpfish-infected *V. anguillarum* J360 (5 days post-infection at 10 °C). (**C**) Survival of lumpfish intraperitoneally infected with *V. anguillarum* J360.

Naïve cultured lumpfish (≈55 g) were intraperitoneally (ip) infected with *V. anguillarum* J360 to evaluate its virulence (Figure 2A,B). Two groups of 60 lumpfish were injected with 1 × 10^6^ and 1 × 10^7^ CFU/dose, respectively. The control group was mock-infected with PBS, and mortality was monitored until 30 days post-infection (dpi). Mortality began at 2 dpi and reached 100% in both doses at 10 dpi (Figure 2C). Vibriosis clinical signs were observed at 5 dpi, including hemorrhage over the lateral lines, dorsal and/or caudal fins, ventral sucker, vent, mouth, and the operculum. Additionally, infected fish exhibited exophthalmia (Figure 2B).

### 3.4. V. anguillarum J360 Genome Sequencing and Annotation

**Figure 3:**
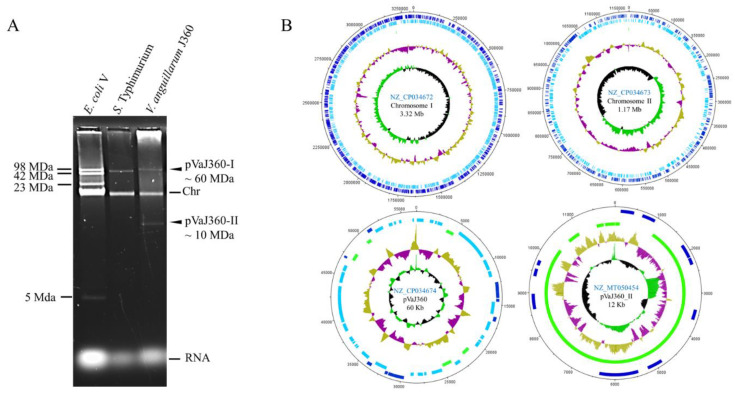
*V. anguillarum* J360 genome visualization. (**A**) *V. anguillarum* J360 plasmid profile in 0.5% agarose using nucleic acids alkaline extraction; *E. coli* V and *S.* Typhimurium UK-1 (χ3761) were used as markers; Chr: Chromosomal band. (**B**) Genome map of *V. anguillarum* strain J360. Chromosome I, chromosome II, large plasmid pVaJ360-I (~60 MDa), and small plasmid pVaJ360-II (~10 MDa) were mapped using DNA plotter. Blue bars represent forward genes; light blue bars represent reverse genes; green bars represent pseudogenes and miscellaneous features. Gold (high) and violet (low) represent G + C content percent; green (high G + C) and black (low G + C) represent skew.

*V. anguillarum* genomic DNA sequenced by PacBio resulted in five contiguous sequences (contigs). The larger contigs corresponded to circularized chromosome-I (3,320,860 bp), chromosome-II (1,171,281 bp), and a large plasmid pVaJ360-I (56,630 bp) with coverage assemblies of 211, 167, and 36 times, respectively. The plasmid profile of *V. anguillarum* J360 indicated that there was also a small plasmid (Figure 3A). Using Illumina reads, we were able to assemble pVaJ306-II (11,995 bp) with a coverage of 226 times. The *V. anguillarum* J360 genome was submitted to NCBI under the BioProject (PRJNA485045) and BioSample (SAMN09781303). The complete genome of *V. angullarum* J360 possesses two chromosomes [chromosome-I (NZ_CP034672) and chromosome-II (NZ_CP034673)], a large plasmid pVaJ360-I (NZ_CP034674), and a small plasmid pVaJ360-II (MT050454), and has an estimated total length of 4.55 Mb and a G + C content of 44.6% (Figure 3B, Table 3). RAST pipeline annotation predicted a total of 441 subsystems and 3149 coding sequences (CDS) for chromosome-I; a total of 88 subsystems and 1143 CDSs for chromosome-II; a total of 2 subsystems and 96 CDSs for the large plasmid pVaJ360-I; and a total of 24 CDS in a single subsystem and 1 total RNAs sequence for the small plasmid pVaJ360-II (Table 4). The NCBI Prokaryote Genome Annotation pipeline showed a total of 4371 genes predicted, a total of 10 (5S), 9 (16S), and 9 (23S) rRNAs, 105 tRNAs, and 4 non-coding RNAs(ncRNAs) for the whole genome (Table 5).

### 3.5. Whole Genome Alignment, Phylogeny, and Synteny

**Figure 4:**
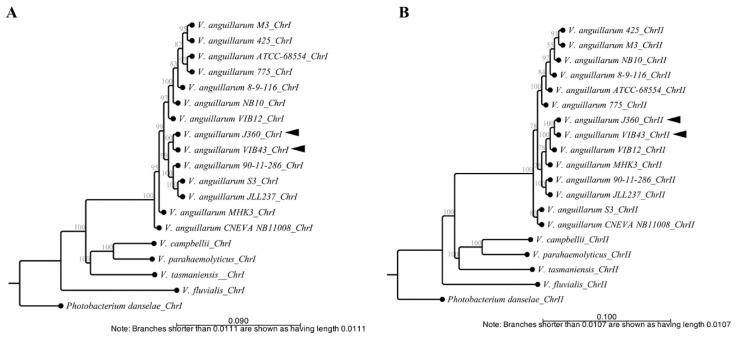

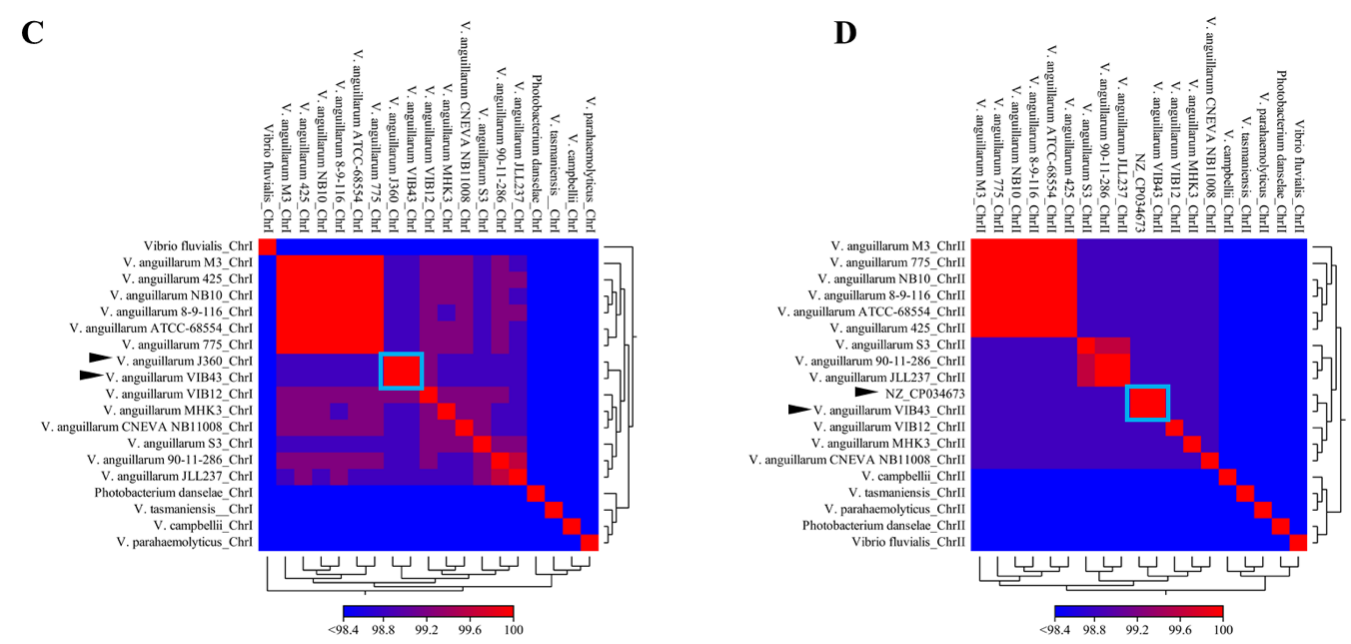
Phylogenetic and comparative genomic analysis of *V. anguillarum* J360. (**A**) Phylogenetic history of *V. anguillarum* J360 chromosome-I. (**B**) Phylogenetic history of chromosome-II. Evolutionary history was inferred using the neighbor-joining method, with a bootstrap consensus of 500 replicates for taxa analysis. Evolutionary distance was computed using the Jukes–Cantor method. All ambiguous positions were removed for each sequence pair (pairwise deletion option). (**C**) Heat map visualization of aligned sequences identity for *V. anguillarum* J360 chromosome-I. (**D**) Heat map visualization of aligned sequences identity for *V. anguillarum* J360 chromosome-II. Whole genome alignments and the phylogenetic analysis involved 18 *Vibrio sp*. listed in Table 1. *Photobacterium damselae* 91–197 as an outgroup. Analysis was performed using CLC workbench v.20 (CLC Bio). Black arrows represent *V. anguillarum* J360 genome and *V. anguillarum* VIB43 as the closest related strain. Light blue square represents the percentage of identity between strains.

**Figure 5:**
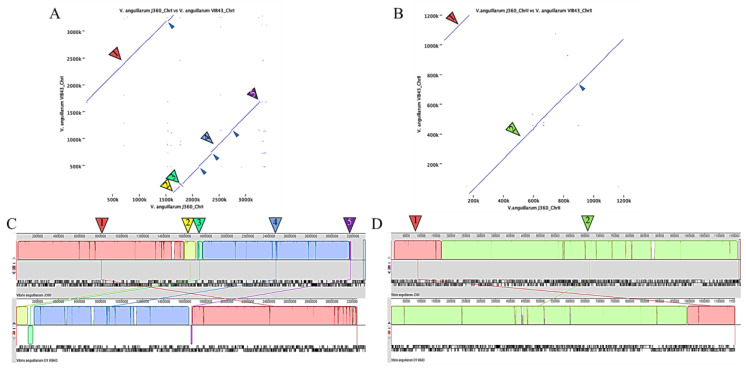
Comparative genome synteny between *V. anguillarum* J360 and *V. anguillarum* VIB43. (**A**) Dot plot analysis for chromosome-I. (**B**) Dot plot analysis for chromosome-II. Numerated arrows represent homologous regions and solid arrows represent inversions. Dot plots were computed using CLC workbench v.20. (**C**) Homologous regions identified as locally colinear blocks (LCBs) (numerated arrows) of chromosome-I. LCB-1 (red); LCB-2 (yellow); LCB-3 (spring green); LC-4 (light blue); LCB-5 (magenta). (**D**) Homologous regions identified as locally colinear blocks (LCBs) (numerated arrows) of chromosome-II. LCB-1 (orange); LCB-2 (green). Small dark-blue arrows indicate genomic gaps or unmatched regions (GGs).

Phylogenetic analysis of *Vibrio* spp. was performed using CLC Bio with only complete genome sequences (Table 1). Phylogenetic analysis of chromosome-I showed that there are three clusters with three or more strains, whereas *V. tasmaniensis*, *V. parahaemolyticus*, and *V. campbellii* clustered together as one and *V. fluvialis* clustered separately. By contrast, *V. anguillarum* species were represented by two clusters plus *V. anguillarum* strains CNEVA, MHK3, VIB12, NB10, and 8-9-116 that clustered separately. *V. anguillarum* J360 was closely related to *V. anguillarum* VIB43 isolated from Scotland, UK (Figure 4A, Table 1). Phylogenetic analysis of chromosome-II indicated four clusters, whereas non-*V. anguillarum* species clustered together, and *V. fluvialis* clustered separately (Figure 4B). Similar to chromosome-I, *V. anguillarum* J360 chromosome-II was closely related to *V. anguillarum* VIB43 (Figure 4B). The same results were observed in the phylogenetic analysis using MEGA X for chromosome-I (Figure S1A) and chromosome-II (Figure S1B). The heat map indicated that there was a high identity between *V. anguillarum* J360 and *V. anguillarum* VIB43 alignments of chromosome-I (Figure 4C) and chromosome-II (Figure 4D), respectively. The ANI analysis for the whole genome alignment indicates a 99.95% for chromosome-I (Figure S2A) and 99.93% for chromosome-II of genome identity (Figure S2B) that support previous observed results. The dot plot visualization matches between *V. anguillarum* J360, and the closest related strain, *V. anguillarum* VIB43, showed that there was a high similarity within the genome. However, two inversion events and genomic gaps (GGs) were identified (Figure 5A,B). The whole genome alignment identified several locally collinear blocks (LCBs), described as conserved segments free from genomic rearrangements [36]. The comparative alignment analysis of each chromosome showed five LCBs in chromosome-I (Figure 5C) and two LCBs in chromosome-II (Figure 5D). Additionally, the LCBs identified in both chromosomes are conserved, and in agreement with the reversion events and GGs identified in the dot plot analysis (Figure 5).

### 3.6. Multi-Locus Sequence Analysis (MLSA) and Phylogeny

We also utilized MLSA to contrast these results with the whole genome analyses. MLSA was computed using the nine housekeeping genes listed in Table S1. Gene sequences were aligned, concatenated, and analyzed. The phylogenetic analysis performed in CLC Bio showed that there were five clusters with at least two strains, whereas *V. anguillarum* J360 clustered alone. This analysis indicated that *V. anguillarum* J360 is closely related to *V. anguillarum* NB10, which clustered with *V. campbelli* and *V. tasmaniensis,* and is distantly related to *V. anguillarum* 775 and *V. anguillarum* M3 (Figure S3A). The same results were observed using MEGA X software (Figure S3B).

### 3.7. Distribution of Genes Associated with Pathogenesis and Environmental Adaptation in V. anguillarum J360

Gene distribution within the *V. anguillarum* J360 chromosomes was determined for virulence and environmental adaptation-related genes (Table 6). Genes associated with iron homeostasis were identified, including ferrous and ferric transport, and regulatory mechanisms. In chromosome-I genes like ferric iron uptake transcriptional regulator (*fur*), *tonB1, tonB2*, and *feoABC* uptake systems were identified. Hemolysis activity-related genes like heme transport (DYL72_00770, DYL72_02835), TonB-dependent hemoglobin receptors (DYL72_17445, DYL72_00730, DYL72_20920), and heme transporters (CcmB and CcmD) were distributed in both chromosomes. Furthermore, five hemolysin-encoding genes (DYL72_01800, DYL72_07805, DYL72_12295, DYL72_17765) were found distributed in both chromosomes, including a thermolabile hemolysin (DYL72_17760) in chromosome-II.

Toxin–antitoxin-associated genes were also identified. We found that *V. anguillarum* J360 possesses five different type II toxin–antitoxin system protein families, such as RelBE/ParDE/DinJ, Txe/YoeB, PhD/YefM, YafQ, and a prevent-host-death system located in chromosome-II. In addition, a toxin precursor gene (*rtxA*), a serine/threonine-protein kinase gene (*hipA*), and four toxin genes (DYL72_00035, DYL72_00045, DYL72_14975, DYL72_14985) were found in chromosome-I, and an antibiotic-resistance gene (*ampC*) was found in chromosome-II.

Metalloprotease coding genes were found in both chromosomes, including a CPBP family intermembrane metalloprotease (DYL72_00295) and a *sprT* family zinc-dependent metalloprotease (DYL72_09830) gene, and three metalloprotease genes (*pmbA*, *tldD*, and *ftsH*) are present in chromosome-I. Additionally, a M6 family domain that possesses metallopeptidase activity was found in chromosome-I. Motility genes were found in chromosome-I, except for flagellar motor brake proteins that were found in chromosome-II. Chemotaxis genes were found on both chromosomes, including 17 genes in chromosome-I and 16 genes in chromosome-II that encode for methyl-accepting chemotaxis proteins. Four copies of *cheV* and five copies of *cheW* were also found, and these are involved in phosphorylation-dependent excitation and methylation-dependent adaptation, respectively. We identified five type IV pilus-associated genes in chromosome-I, and type VI secretion system-related genes in both chromosomes, including six copies of *tssI*, and two operons encoding *tssBCEFG* and *tssKJHFE2*. Two secretion system families were identified, including the Hcp type VI secretion system family effector and DotU family type IV and type VI secretion system-related proteins. A single quorum-sensing-associated gene was found in chromosome-II, which is a quorum-sensing autoinducer synthase. Transcriptional regulators were found in chromosome-I, such as *lysR*, *cysB*, *nhaR*, and *hfq,* and *luxR* was present in both chromosomes.

### 3.8. Genomic Islands (GIs)

**Figure 6:**
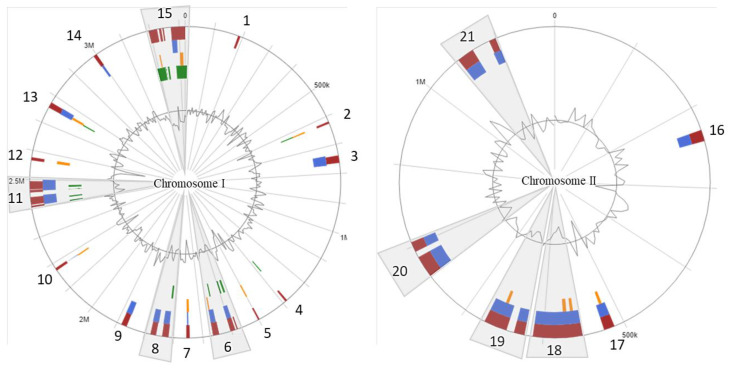
*V. anguillarum* J360 genomic islands (GIs). Genomic islands (GIs) detected in chromosome-I; genomic islands (GIs) detected in chromosome-II. Red bars represent GIs detected using 3 different packages; blue bars represent GIs detected with the SIGI-HMM package; orange bars represent GIs detected with the IslandPath-DIMOB package; green bars represent GIs detected with the IslandPick package.

Twenty-one putative GIs were identified within the chromosomes; 15 GIs in chromosome-I and 6 GIs in chromosome-II, respectively (Figure 6; Supplementary file 1 and 2). The GIs size ranged from 5 to 73.4 kb with a total of 724 genes. The largest genomic island (GI15) (Figure 6) consisted of 147 genes (predicted by at least one of the three software used, see Method section), and was flanked by two site-specific integrase genes and a zinc ribbon-domain protein.

Genes encoding for integrases, porins, transposases, and iron transport were found among the GIs. Genes such as phosphonate C-P lyases, associated with the cleavage of carbon-phosphorus compounds for organic phosphorus reservoir in marine bacteria [44], zinc ribbon domain proteins, AbrB/MazE/SpoVT DNA-binding domains, a ParA domain, for a GCN5-related N-acetyltransferase (GNAT) associated with regulatory post-transcriptional acetylation [45], two site-specific integrases, and a MasF transcriptional regulator related to toxin/anti-toxin systems (Supplementary file 1) were identified in these GIs. GI5 is the smallest genomic island identified in *V. anguillarum* J360 (Figure 6), which possesses five unique genes that encode for acetyltransferase, acyltransferase, formyltransferase, asparagine synthase, and one hypothetical protein (Supplementary File 1).

### 3.9. V. anguillarum Large Plasmids Analysis

The plasmid profile indicates that *V. anguillarum* J360 harbors one large plasmid and one small plasmid (Figure 3A). The large plasmid pVaJ360-I (~60 kb) has genes that encode for integrases, DNA-binding proteins, peptidases, site-specific integrases, resolvase, mobile elements, and pro-phages. Comparative analysis showed that pVaJ360-I is not related to *V. anguillarum* virulent plasmids such as pJM1 (strain 775); p65 (strain ATCC-68554); pJM1-like plasmid, p67 (strain NB10); and p15 (strain VIB43) (Figure S5A). The ANI analysis of these plasmids demonstrated that pVaJ360-I does not have a percentage of identity with pJM1 or pJM1-like plasmids (Figure S5B), suggesting that pVaJ360 is not a virulent plasmid.

The small plasmid pVaJ360-II (~12 kb) has only 10 CDSs that encode for hypothetical proteins, transposases, mobile elements, a transcriptional regulator LysR, a tRNA Glu, and additionally 14 miscellaneous features. BLASTn analysis indicates that this plasmid has no similarities with the pJM1 or pJM1-like plasmids described above.

## 4. Discussion

Lumpfish across hatcheries and deployment sites frequently show signs of systemic bacterial infection, including skin lesions, gill hemorrhages, and bacterial aggregations in lymphoid organs (i.e., spleen, liver, head kidney) [10]. In the United Kingdom, Iceland, and Norway, several bacterial outbreaks have been reported in lumpfish hatcheries and at cage sites, and the most frequent pathogen detected is *V. anguillarum* [46]. Thus, it is not surprising that this pathogen was found to be present in Atlantic Canada.

*V. anguillarum* serotypes O1, O2, and O3 are the most prevalent strains among the 23 serotypes currently described [18,19]. Agglutination assays indicated that *V. anguillarum* J360 is O2, similar to other *V. anguillarum* strains isolated from lumpfish infections in the North Atlantic [46]. The biochemical profile obtained using API20NE showed that *V. anguillarum* J360 was able to reduce sugars, urea, and produce indole, suggesting a 99% possibility for *V. fluvialis* (Table S3). Although the biochemical profile did not identify *V. anguillarum* J360, its phenotypic characterization is consistent with other *V. anguillarum* isolates [25,26], except that *V. anguillarum* J360 was positive for urease (Table S4). This result is coincident with the presence of the urease encoding gene in chromosome-II (DYL2_19555). In addition, *V. anguillarum* J360 was not able to grow in TCBS-selective media, suggesting susceptibility to bile salts or a poor adaptation to the culture medium (Table 2) [47].

*V. anguillarum* J360 showed a thermo-inducible α-hemolysin activity at 28 °C, but no hemolytic activity was observed at temperatures below 15 °C (Figure 1E). Hemolytic activity is an important virulence factor for *V. anguillarum* [48]. For instance, severe hemorrhages are a typical clinical sign of *V. anguillarum* infection in fish, including lumpfish [46]. Coincidently, in the current study, *V. anguillarum* J360 was shown to be highly virulent in lumpfish, and infected lumpfish displayed severe hemorrhagic symptoms at 5 dpi (Figure 2B), similar to other strains described in Marco-Lopez et al. 2013. Koch’s postulates for *V. anguillarum* J360 showed that lumpfish infected with 1 × 10^6^ and 1 × 10^7^ CFU/dose reached 100% mortality within 10 dpi at 10 °C (Figure 2C). In addition, *V. anguillarum* was re-isolated from the spleen, liver, and head-kidney, confirming Koch’s postulates. The original *V. anguillarum* outbreak in cultured lumpfish and the infection assays in the current study showed similar clinical signs (Figure 2B). *V. anguillarum* hemolytic activity was evident during infection. However, the lumpfish is a cold-water fish typically cultured between 6 and 12 °C [49]. These results contradicted with the *V. anguillarum* thermo-inducible hemolytic activity at 28 °C (Figure 1E). Perhaps, internal fish conditions (e.g., innate and adaptive immunity) triggered *V. anguillarum* hemolytic activity. These results suggest that its regulatory mechanisms need further analysis.

*V. anguillarum* J360 possesses two chromosomes, a large plasmid and a small plasmid (Figure 3 and Table 4). *Vibrio* spp. and *V. anguillarum* genomes selected for phylogenetic and comparative genomics analysis possess two chromosomes and one large plasmid (Table 1). Typically, serotype O1 harbors a virulent plasmid, called pJM1 or pJM1-like, and serotypes O2 and O3 strains possess a nonvirulent large plasmid [50,51] or they do not harbor large plasmids [20,37]. *V. anguillarum* J360 is a O2 serotype that does not harbor a virulence plasmid (Figure S5), suggesting that this strain could increase its virulence if a virulence plasmid is acquired.

The total genome size of *V. anguillarum* J360 is 4,561,566 bp (Table 4), which is larger than the currently available *V. anguillarum* genomes (Table 1). This may suggest that *V. anguillarum* J360 may have acquired genetic material through horizontal gene transfer and/or adapted to its lumpfish host or to environmental conditions in Atlantic Canada.

Phylogenetic distance based on the whole genome alignment analysis of chromosome-I and chromosome-II showed that *V. anguillarum* J360 is closely related to *V. anguillarum* VIB43, and distantly related to *V. anguillarum* VIB12 (Figure 4A,B). Interestingly, *V. anguillarum* VIB43 and VIB12 were isolated from the same host species, sea bass (*Dicentrarchus labrax*), but from different locations. *V. anguillarum* VIB43 was isolated in Scotland and *V. anguillarum* VIB12 was isolated in the Mediterranean Sea [20]. The ANI analysis between *V. anguillarum* J360 and *V. anguillarum* VIB43 showed a 99.93% identity for chromosome-I and 99.95% for chromosome-II (Figure 4C,D and Figure S2A,B), which suggest that these two strains share a common ancestor.

Additionally, the whole genome phylogenetic analysis showed that *V. anguillarum* J360 and VIB43 are not closely related to *V. anguillarum* 775, M3, and NB10, which is contradictory to previous MLSA studies that indicated that *V. anguillarum* VIB43 and VIB12 are closely related to those *V. anguillarum* strains [20,50]. The MLSA computed the phylogenetic stress based on concatenated conserved sequences [51,52]. In this study, we used nine conserved housekeeping genes, including 16S rRNA, *ftsZ*, *gapA*, *gyrB*, *mreB*, *pyrH*, *recA*, *rpoA*, and *topA* (Table S1). In contrast to the whole genome phylogenetic analysis, the MLSA showed that *V. anguillarum* J360 clusters alone, and the closest related strain is *V. anguillarum* NB10 isolated from rainbow trout (*Oncorhynchus mykiss*) in the Gulf of Bothnia, Sweden [6], which is distantly related to *V. anguillarum* 775 and M3 strains (Figure S3, Table 1). *V. anguillarum* M3 was isolated from Japanese flounder (*Paralichthys olivaceus*) in Shandong, China, and classified as closely related to *V. anguillarum* 775 isolated from Coho salmon (*Oncorhynchus kisutch*) in the Pacific coast of USA [7,8]. By contrast, MLSA phylogenetic analysis based only on the 16S rRNA gene showed that *V. anguillarum* NB10 is closely related to *V. anguillarum* M3 [6].

In contrast to the MLSA analysis, the whole genome phylogenetic analysis of *V. anguillarum* strains is in concordance with the geographic origin of the strain isolation. For instance, according to the whole genome phylogenetic analysis, *V. anguillarum* J360 and *V. anguillarum* VIB43, both isolated in the North Atlantic Ocean, are highly related, and closely related to *V. anguillarum* strains 90-11-286, S3, and JLL237 isolated in Finland (Table 1; Figure 4). Actually, these geographic locations are a natural habitat for lumpfish populations [53].

In contrast to the whole genome phylogenetic analysis, the MLSA uses protein-coding genes, which evolved at a slow but constant rate, and it could have better resolution, especially at the species level [51,52]. However, the selection and number of coding genes, and alignment method, are variable for MLSA. We found that the phylogenetic analysis using whole genomes is more reliable than MLSA, and the robustness of our analyses showed to be consistent with two different software. In addition, whole genome analyses allow homologous regions, deletion, translocation, and inversion events to be identified.

Genome alignment and synteny analysis between *V. anguillarum* J360 and *V. anguillarum* VIB43 showed a high similarity within the chromosome sequences, but inversions and unmatched regions were also observed (Figure 5A,B). This suggests that homologous recombination events play an important role in *V. anguillarum* evolution, perhaps influenced by insertion sequence (IS) elements such as chromosomal integrons or “super integrons” (SIs) describing *Vibrio* spp. and several Gram-negative species [20]. We determined that there are five LCBs in chromosome-I (Figure 5C) and two LCBs in chromosome-II (Figure 5D) shared between *V. anguillarum* J360 and VIB43. Further analysis revealed that all the LCBs present in chromosome-I have small inversion events (Figure 5A,C). In addition, we found that LCBs-1 and -4 have genome gaps (GGs) or unmatching regions in both strains (Figure 5A and Figure S4A). The GGs identified in LCB-1 of *V. anguillarum* J360 chromosome-I are not present in *V. anguillarum* VIB43 LCB-1 (Figure S4A). These identified GGs possess several genes that encode for IS families transposases (IS66, ISL3, IS3, IS5) and site-specific integrases previously described in the *V. anguillarum* VIB43 genome, and with high similarity to *V. anguillarum* NB10, 775, and ATCC-6855 genomes [6,20]. We found that the unique GGs in LCB-1 of *V. anguillarum* J360 possess genes related to iron uptake and iron homeostasis (Figure S4A), suggesting that these genes could be acquired by horizontal gene transfer.

In *V. anguillarum* J360 chromosome-II, two GGs were identified in LCB-1 and LCB-2 (Figure S4B), and both GGs have an IS630-like element belonging to the ISVa15 transposase family. This IS630-like element is not present in *V. anguillarum* VIB43 LCBs. According to the description of Holm et al. (2018), ISVa3–ISVa20 are new insertion sequence (IS) elements in the *V. anguillarum* genomic repertory that are responsible for the divergency within strains. This suggests that *V. anguillarum* J360 and *V. anguillarum* VIB43 could be derived from a common ancestor and adapted to local environmental conditions and host species.

Pathogenesis-associated genes were found in both chromosomes, but chromosome-I harbors most of the virulence genes and their respective transcriptional regulators (Table 6). No virulence-associated genes were found in the large plasmid pVaJ360-I or in the small plasmid pVaJ360-II. The *V. anguillarum* virulence plasmid pJM1 possesses intrinsic virulence genes associated with iron uptake, like anguibactin biosynthesis (*angA-angE*, *vabA-E*) and anguibactin transport (*fatA-fatD*) [8,54,55]. By contrast, all the *V. anguillarum* J360 iron homeostasis-related genes are in its chromosomes. For instance, genes related to ferric-anguiobactin and siderophore uptake (e.g., *exbB* and *exbD2*, respectively) are present in chromosome-I. Comparative genomic analyses showed that the large virulent plasmids pJM1, P67-NB10, and p65-ATCC have high similarity (Figure S5A,B). However, the large plasmid pVaJ360-I and the small plasmid pVaJ360-II of *V. anguillarum* J360 do not present similarity (Figure S5A) or identity (Figure S5B) with other reported plasmid sequences, nor possess virulence-associated genes. This suggests that *V. anguillarum* J360 does not harbor plasmids previously described in *V. anguillarum,* including serotype O2 strains [50].

Hemolysins are important virulence factors for *V. anguillarum* species, and contribute to its attachment, tissue colonization, and iron homeostasis, thus increasing its pathogenicity [56,57]. *V. anguillarum* J360 has four hemolysin genes, and these are consistent with the hemorrhagic clinical signs observed in lumpfish during the infection assays (Figure 2B). In addition, a thermolabile hemolysin gene is present in chromosome-II, which can be related to the thermo-inducible hemolytic phenotype of *V. anguillarum* J360 (Figure 1E).

The resistance of *V. anguillarum* J360 to ampicillin (Table 2) relates to the presence of a class C beta-lactamase (*ampC*)-encoding gene in chromosome-II. Metalloproteases such as *pmbA*, *tldD*, and *ftsH* genes, which are associated with carbon storage, hydrolysis of peptide bonds, and virulence, were also identified.

*V. anguillarum* J360 does not possess some of the virulence genes present in *V. anguillarum* strains isolated from the Pacific coasts, including the metalloproteases *empA* and *prtV* [8,58]. Nonetheless, *V. anguillarum* J360 harbors a M6 family metalloprotease (DYL72_17780) similar to the *prtV* gene, associated to gelatinase activity (Table S3).

In addition, genes that encoded for secreted enzymes such as phospholipase and lipases were found in chromosome-II, which correlates with the lipase (C_14_)-positive phenotype observed in the enzymatic profile (Table S3). *V. anguillarum* J360 possesses several genes associated with flagella and motility, such as the operons *fliRQPONMLKJIHGFE* (DYL72_03140-DYL72_03205), *flgLKJIHGFEDCB* (DYL72_03685-DYL72_03735), and *motYBA* (DYL72_12660, DYL72_00275, DYL72_12090) located in chromosome-I, which is consistent with the mot^+^ phenotype of *V. anguillarum* J360 (Table 2).

Virulence factors present in other *V. anguillarum* genomes (e.g., *V. anguillarum* 775 and *V. anguillarum* M3) were not identified in the *V. anguillarum* J360 genome. These include mannose-sensitive hemagglutinin type 4 pilus (MHSA) [8,54], *vstA*-*vstH* genes for a Type VI secretion system [55], and *virA*-*virB* genes related to lipopolysaccharides synthesis [58].

In concordance with the locations of the virulence factors, transcriptional regulators such as *luxR*, *lysR*, *cysB*, *nhaR*, and *hfq* were mostly located in chromosome-I, and an additional copy of *luxR* was found in chromosome-II. LuxR belongs to a transcriptional activators family, that together with an N-(3-oxodecanoyl)-L-homoserine lactone (ODHL), mediates the signal transduction mechanisms of quorum-sensing genes such as *luxICDABE* operon [59]. The duplication of *luxR* in *V. anguillarum* J360 suggests that quorum-sensing plays an important role in the biology of this strain.

Genomic Islands (GIs) have been identified in *V. anguillarum* species, for instance, *V. anguillarum* NB10 possesses 29 GIs [6], *V. anguillarum* 775 possesses 10 GIs [8], and *V. anguillarum* J360 has 21 GIs (Figure 6). We found that GI-19 in chromosome-I (2,994,299..3,011,196 nt) of *V. anguillarum* NB10 [6] has some homologous regions with *V. anguillarum* J360 GI-14 in chromosome-I (2,988,473..3,000,617 nt). In addition, we determined that GI-25 (546,066..578,220 nt) and GI-26 (639,559..674,888 nt) in chromosome-II of *V. anguillarum* NB10 [6] share similar genetic context with GI-18 (552,330..612,508 nt) and GI-19 (622,682..673,456 nt) in chromosome-II of *V. anguillarum* J360, respectively. However, the low similarity between GIs of *V. anguillarum* J360 and *V. anguillarum* NB10 suggests a relatively distant relationship, consistent with the whole genome phylogenetic analysis (Figure 4).

In contrast, 19 GIs were identified in *V. anguillarum* VIB43 (Figure S6, Supplementary files 3 and 4), which showed high similarities with the GIs founded in *V. anguillarum* J360. For instances, GI-1, GI-4, and GI-12 present in *V. anguillarum* VIB43 chromosome-I (Figure S6) possess several homologous regions with GI-8, GI-10, and GI-3 of *V. anguillarum* J360 chromosome-I (Figure 6), respectively (Supplementary files 1 and 3). In addition, GI-6 shared homologous regions with GIs 11 and 12; however, all these regions are flanked by IS66 or IS66-like family transposases (ISVa9, ISVa15, ISVa11), suggesting that these regions are hot spots for recombination events (Supplementary files 1 and 3). GI-12 of *V. anguillarum* VIB43 and GI-3 of *V. anguillarum* J360 are highly conserved (Supplementary 1 and 3). Similar results were observed in chromosome-II. GI-15 and GI-16 of *V. anguillarum* VIB43 (Figure S6) showed several homologous regions with GI-16 and GI-17 of *V. anguillarum* J360 (Figure 6), respectively (Supplementary files 2 and 4). GI-17 of *V. anguillarum* VIB43 has several homologous regions with GIs-18 and GI-19 of *V. anguillarum* J360 (Supplementary files 2 and 4). The homologous regions between GIs-17 of *V. anguillarum* VIB43, and GI-18 and GI-19 of *V. anguillarum* J360 encode for several virulence factors like lipocalin, Hcp tube protein (T6SS), interferase toxin, damage-inducible protein J, secretion proteins, and toxins. These homologous regions are flanked by several IS elements (Supplementary files 2 and 4), which indicates that these three GIs are genomic pathogenic islands, perhaps acquired through horizontal gene transference [60]. These results support the hypothesis that these IS elements (IS66, ISL3, IS3, IS5)^6^ are responsible for the genomic gaps (GGs) and genomic rearrangements previously mentioned, as well as support the 0.05–8% of genomic differences observed at the identity analyses.

## 5. Conclusions

In this study, the complete genome of *V. anguillarum* J360 serotype O2 isolated from infected cultured lumpfish in Newfoundland, Canada was reported. *V. anguillarum* J360 has a larger genome size (4,549,571 bp) as compared to other available *V. anguillarum* genomes. The *V. anguillarum* J360 genome has genes related to antibiotic resistance, hemolysin activity, gelatinase, and lipases that play a major role in virulence. *V. anguillarum* J360 was shown to be closely related to the *V. anguillarum* VIB43 strain isolated in Scotland, UK, from sea bass. Comparative genomics revealed that five LCBs are shared between *V. anguillarum* J360 and *V. anguillarum* VIB43 chromosome-I, and two LCBs are shared in chromosome-II. Twenty-one genomic islands (GIs) were identified within the chromosomes of *V. anguillarum* J360. Similar GIs identified in *V. anguillarum* J360 were found in *V. anguillarum* VIB43 chromosomes, and this is consistent with the whole genome phylogenetic analysis. *V. anguillarum* J360 has virulence-associated genes in both chromosomes but does not harbor a virulent plasmid.

## Figures and Tables

**Table 1:** Geographic origin and hosts species of *Vibrio anguillarum* isolates.

Species and Serotype	Geographic Origin, Host Species	Genome Size (bp)	Accession Numbers	References
*V. anguillarum* 775/O1	USA, Pacific coast/*Oncorhynchus kisutch*	4,117,056	CP002284/5	[8]
*V. anguillarum* M3/O1	China, Shandong/*Paralichthys olivaceus*	4,117,885	CP006699/700	[7]
*V. anguillarum* NB10/O1	Sweden, Baltic Sea/*Oncorhynchus mykiss*	4,373,835	LK021130/29	[6]
*V. anguillarum* VIB12/O2	Greece/*Dicentrarchus labrax*	4,897,690	CP023310/11	[20]
*V. anguillarum* VIB43/O1	Scotland, UK/*Dicentrarchus labrax*	4,407,865	CP023054/5	[20]
*V. anguillarum* CNEVA/O3	France/*Dicentrarchus labrax*	4,256,429	CP022103/4	[20]
*V. anguillarum* MHK3/O1	China/Flounder	4,015,925	CP022468/9	-
*V. anguillarum* 425	China/Sea bass	4,373,373	CP020533/4	-
*V. anguillarum* 87-9-116/**O1	Finland/*Salmo salar*	4,338,125	CP021980/1	[20]
*V. anguillarum* JLL237/O1	Denmark/*Oncorhynchus mykiss*	4,286,989	CP022101/2	[20]
*V. anguillarum* ATCC-68554/O1	USA, Pacific coast/*Oncorhynchus kisutch*	4,141,906	CP023209/8	[20]
*V. anguillarum* 90-11-286	Denmark/Farm-water sample	4,342,224	CP011460/1	[37]
*V. anguillarum* S3/O1	Denmark/*Oncorhynchus mykiss*	4,272,973	CP022099/100	[20]
*V. campbelli* ATCC 25920	USA, Hawaii/Seawater isolate	5,178,103	CP015863/4	[38]
*V. fluvialis* ATCC 33809	Bangladesh/*Homo sapiens*	4,827,733	CP014034/5	[39]
*V. parahaemolyticus* ATCC 17802	Japan/Seawater isolate	5,152,461	CP014046/7	[40]
*V. tasmaniensis* LGP32	France/*Crassostrea gigas*	4,974,818	FM954972/3	[41]
*Photobacterium damselae* 91-197	USA/Hybrid Striped Bass (*Morone* sp.)	4,293,175	AP018045/6	[35]

**Table 2:** Phenotypic characteristics of *V. anguillarum* J360.

Characteristic	*Vibrio anguillarum* J360
Growth at:	
4 °C	+
15 °C	+
28 °C	+
37 °C	−
LB NaCl 0%	−
LB NaCl 0.5%	+
LB NaCl 2%	+
Plate Count Agar 50% seawater	+
TCBS	−
Motility	+
Fimbria Type I	+
Siderophores synthesis	+
Catalase	+
Oxidase	+
Antibiogram:	Halo diameter (mm)
Tetracycline (10 mg/mL)	31 (Susceptible)
Oxytetracycline (30 mg/mL)	34 (Susceptible)
Ampicillin (10 mg/mL)	0 (Resistant)
Sulfamethoxazole (25 mg/mL)	33 (Susceptible)
Chloramphenicol (30 mg/mL)	35 (Susceptible)
Colistin sulphate (10 mg/mL)	15 (Susceptible)
Oxalinic acid (2 mg/mL)	39 (Susceptible)
O-129	39 (susceptible)

**Table 3:** *V. anguillarum* J360 genome summary.

Labels	Size (Mb)	Topology	RefSeq ID	INSDC Identifier
Chromosome-I	3.32	Circular	NZ_CP034672	CP034672
Chromosome-II	1.17	Circular	NZ_CP034673	CP034673
Plasmid pVaJ360-I	0.06	Circular	NZ_CP034674	CP034674
Plasmid pVaJ360-II	0.012	Circular	NZ_MT050454	MT050454

**Table 4:** Rapid annotation subsystem technology (RAST) *V. anguillarum* J360 annotation summary.

	Chromosome-I	Chromosome-II	Plasmid pVaJ360-I	Plasmid pVasJ360-II
Genome size (bp)	3,320,860	1,172,081	56,630	11,995
G+C content (%)	44.6	44.1	43.7	47
Number of subsystems	441	88	2	1
Number of coding sequences	3149	1143	96	24
Number of RNAs	129	5	-	1

**Table 5:** NCBI prokaryotic Genome Annotation pipeline *V. anguillarum* J360 genome annotation summary.

Attribute	Data Provided
Annotation pipeline	NCBI
Annotation method	Best placed reference protein set; GeneMarks v4.6
Genes (total)	4371
CDSs (total)	4234
Genes (coding)	3966
Genes (RNA)	137
rRNAs	10, 9, 9 (5S, 16S, 23S)
Complete rRNAs	10, 9, 9 (5S, 16S, 23S)
tRNAs	105
ncRNAs	4
Pseudogenes (total)	268
Pseudogenes (ambiguous residues)	0 of 268
Pseudogenes (frameshifted)	105 of 268
Pseudogenes (incomplete)	163 of 268
Pseudogenes (internal stop)	45 of 268
Pseudogenes (multiple problems)	41 of 268

**Table 6:** Predicted genes associated with subsystems of pathogenesis and environmental adaption.

Gene Subsystem Category and Name	Presence/Absence of Gene in *V. anguillarum* J360		GenBank Accession N°
	Chromosome-I	Chromosome-II	
Iron transport and regulation			
iron-regulated protein A	X		DYL72_00705
*tonB2*, *exbD2*	X		DYL72_00755, DYL72_00745
*tonB1*, *exbB*, *exbD1*	X		DYL72_00260, DYL72_00265, DYL72_00270
*fur*	X		DYL72_03070
iron ABC transport permease	X		DYL72_00175, DYL72_00765
Heme transport			
Ton-B-dependent hemoglobin receptor	X	X	DYL72_17445, DYL72_00730, DYL72_20920
*hutXZ*	X		DYL72_00735, DYL72_00740
heme ABC transporter protein	X		DYL72_00770, DYL72_02835
heme exporter protein *ccmBD*	X		DYL72_02830, DYL72_02840
Ferrous and ferric transport			
ferric ABC transporter	X		DYL72_00180
*feoABC*	X		DYL72_02945, DYL72_02940, DYL72_02935
Ferrichrome			
*fhuACBD*	X		DYL72_06770, DYL72_10590
Hemolysins			
hemolysin genes	X	X	DYL72_01800, DYL72_07805, DYL72_12295, DYL72_17765
thermolabile hemolysin		X	DYL72_17760
Toxins-associated genes			
toxins and pseudogenes	X		DYL72_00035, DYL72_00045, DYL72_14975, DYL72_14985
*rtxA*, *hipA*	X		DYL72_01180, DYL72_03440
*ampC*		X	DYL72_17850
type II toxin–antitoxin system RelBE/ParDE/DinJ family		X	DYL72_18560, DYL72_18565, DYL72_18715, DYL72_18750, DYL72_19140, DYL72_19250, DYL72_19960
type II toxin–antitoxin system prevent-host-death family antitoxin		X	DYL72_18805
Txe/YoeB family addiction module		X	DYL72_18810
type II toxin–antitoxin system Phd/YefM family antitoxin		X	DYL72_19255
type II toxin–antitoxin system YafQ family toxin		X	DYL72_19965
toxin–antitoxin system subunit antitoxin		X	DYL72_20370
Metalloproteases			
CPBP family intramembrane metalloprotease	X		DYL72_00295
*pmbA*, *tldD*, *ftsH*	X		DYL72_05300, DYL72_09550, DYL72_10735
SprT family zinc-dependent metalloprotease	X		DYL72_09830
M6 family metalloprotease domain-containing protein		X	DYL72_17780
Secreted enzymes			
phospholipase gene		X	DYL72_16230
lipase		X	DYL72_17465, DYL72_18050, DYL72_20375
Motility and Chemotaxis			
*fliRQPONMLKJIHGFE, fliSTD, fliL*	X		DYL72_03140-DYL72_03205, DYL72_03225-DYL72_03235, DYL72_08330
*motYA*, *motB*	X		DYL72_12660, DYL72_00275, DYL72_12090
*flhFAB*	X		DYL72_02900, DYL72_02905, DYL72_03135,
*flag*, *flaCA*	X		DYL72_03240, DYL72_03670, DYL72_03675
flagellin	X		DYL72_03245, DYL72_03250, DYL72_03255
*flgLKJIHGFEDCB*, *flgAMNP*	X		DYL72_03685-DYL72_03735, DYL72_03750-DYL72_03765
flagellar basal-body protein	X		DYL72_03775
*pomA*	X		DYL72_12085
flagellar brake protein		X	DYL72_17355, DYL72_20195
methyl-accepting chemotaxis protein	X (17)	X (16)	
chemotaxis response regulator protein-glutamate methylesterase	X	X	DYL72_00595, DYL72_02870, DYL72_16590,
*cheD*, *cheW, cheA, cheV, cheY, cheX, cheC*	X	X	DYL72_00600, DYL72_00610, DYL72_00620, DYL72_01045, DYL72_02885, DYL72_09235, DYL72_20035
*cheV2*, *cheW2, cheW3, cheA2, cheV3, cheD2, cheW4, cheW5, cheA3, cheV4*	X	X	DYL72_02435, DYL72_02855, DYL72_02860, DYL72_02875, DYL72_03745, DYL72_16595, DYL72_16610, DYL72_16615, DYL72_16620, DYL72_21345
chemotaxis protein	X	X	DYL72_00635, DYL72_16275, DYL72_16460
chemotaxis protein methyltransferase	X		DYL72_03740
Type IV pilus			
*pilQ*, *pilW, pilM, pilT, pilB*	X		DYL72_05800, DYL72_01100, DYL72_05820, DYL72_09795, DYL72_10350
Type VI secretion system (T6SS)			
*tssI*, *tssBCEFG, tssJ, tssK tssH, tssAM*		X	DYL72_16415, DYL72_17000—DYL72_17020, DYL72_17030, DYL72_17045, DYL72_17055, DYL72_17070, DYL72_17075,
*tssI2*, *tssM2, tssKJHFE2, tssC2, tssI3, tssI4, tssI5, tssI6*	X	X	DYL72_17085, DYL72_21115, DYL72_00885, DYL72_00895, DYL72_00900, DYL72_00930, DYL72_00940, DYL72_00945, DYL72_00955, DYL72_00960, DYL72_00975, DYL72_02685, DYL72_10015
*tagHO*		X	DYL72_17025, DYL72_17065
Hcp family type VI secretion system effector	X	X	DYL72_16420, DYL72_18695, DYL72_21120, DYL72_02690, DYL72_10010
type VI secretion system tube protein Hcp	X		DYL72_00965
DotU family type IV/VI secretion system protein	X	X	DYL72_17050, DYL72_00890
type VI secretion protein	X	X	DYL72_17080, DYL72_00950, DYL72_00970, DYL72_00985
type VI secretion system PAAR protein	X	X	DYL72_21095, DYL72_02650, DYL72_02660, DYL72_10045,
type VI secretion protein VasB-1	X		DYL72_00935
Quorum sensing			
quorum-sensing autoinducer synthase		X	DYL72_17995
Regulators			
transcriptional regulator LuxR	X	X	DYL72_06050, DYL72_21285
transcriptional regulator LysR	X		DYL72_03060, DYL72_09990
transcriptional regulator XRE	X		DYL72_10570
*cysB*	X		DYL72_13470
*nhaR*	X		DYL72_10935
*hfq*	X		DYL72_09115

## Supplementary Materials

The following are available online at https://www.mdpi.com/2076-2607/8/11/1666/s1, Figure S1: Evolutionary taxa relationship of *V. anguillarum* chromosome-I and chromosome-II, Figure S2: Average nucleotide identity (ANI) comparison of *V. anguillarum* whole genome alignment, Figure S3: Phylogenetic analysis of *V. anguillarum* using Multi Locus Sequence Analysis (MLA), Figure S4: Comparative whole genome alignment closeup between *V. anguillarum* J360 and *V. anguillarum* VIB43, Figure S5: Comparative analysis *V. anguillarum* J360 large plasmid pVaJ360-I, Figure S6: *V. anguillarum* VIB43 genomic islands (GIs), Table S1: Enzymatic profile of *V. anguillarum* J360 using the API ZYM system, Table S2: Enzymatic profile of *V. anguillarum* J360 using API20NE, Table S3: Enzymatic profile of *V. anguillarum* J360 using API20E, Table S4. Housekeeping genes used for MLSA. Supplementary file S1: Genomic islands *V. anguillarum* J360 chromosome-1; Supplementary file S2: Genomic islands *V. anguillarum* J360 chromosome-2; Supplementary file S3: Genomic islands *V. anguillarum* VIB43 chromosome-1; Supplementary file S4: Genomic islands *V. anguillarum* VIB43 chromosome-2.
