# Supplementary material for: Comparative Genomics Analysis of Vibrio anguillarum Isolated from Lumpfish (Cyclopterus lumpus) in Newfoundland Reveal Novel Chromosomal Organizations
Source: Microorganisms. 2020 Oct 27;8(11):1666. doi: 10.3390/microorganisms8111666 (PMC7716436; doi:10.3390/microorganisms8111666)
Supplement: Supplementary file 1 [file microorganisms-08-01666-s001.zip › Supplementary/Supplementary figures.docx]

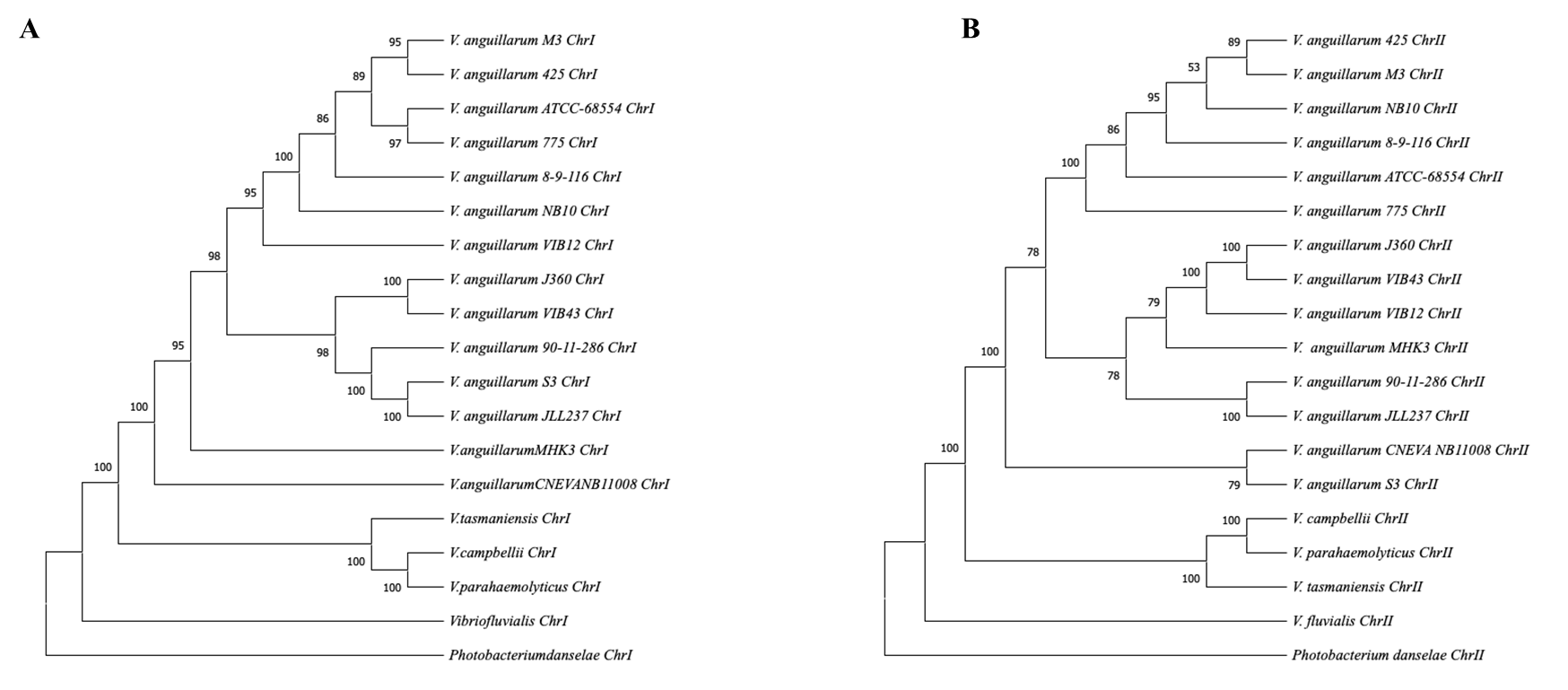


**Figure S1. Evolutionary taxa relationship of *V. anguillarum* chromosome-I and chromosome-II. A.** *V. anguillarum* chromosome-I evolutionary relationships; **B.** *V. anguillarum* chromosome-I evolutionary relationships. Evolutionary history was calculated using Neighbor-Joining method with a bootstrap consensus tree (500 replicates). Evolutionary distances were computed using the Jukes-Cantor method. and are in the units of the number of base substitutions per site. Ambiguous positions were removed for each sequence pair (pairwise deletion option). There was a total of 1,322,945 positions in the final dataset for chromosome-I and 310,515 positions in the final dataset of chromosome-II. Evolutionary analyses were conducted in MEGA X.

**
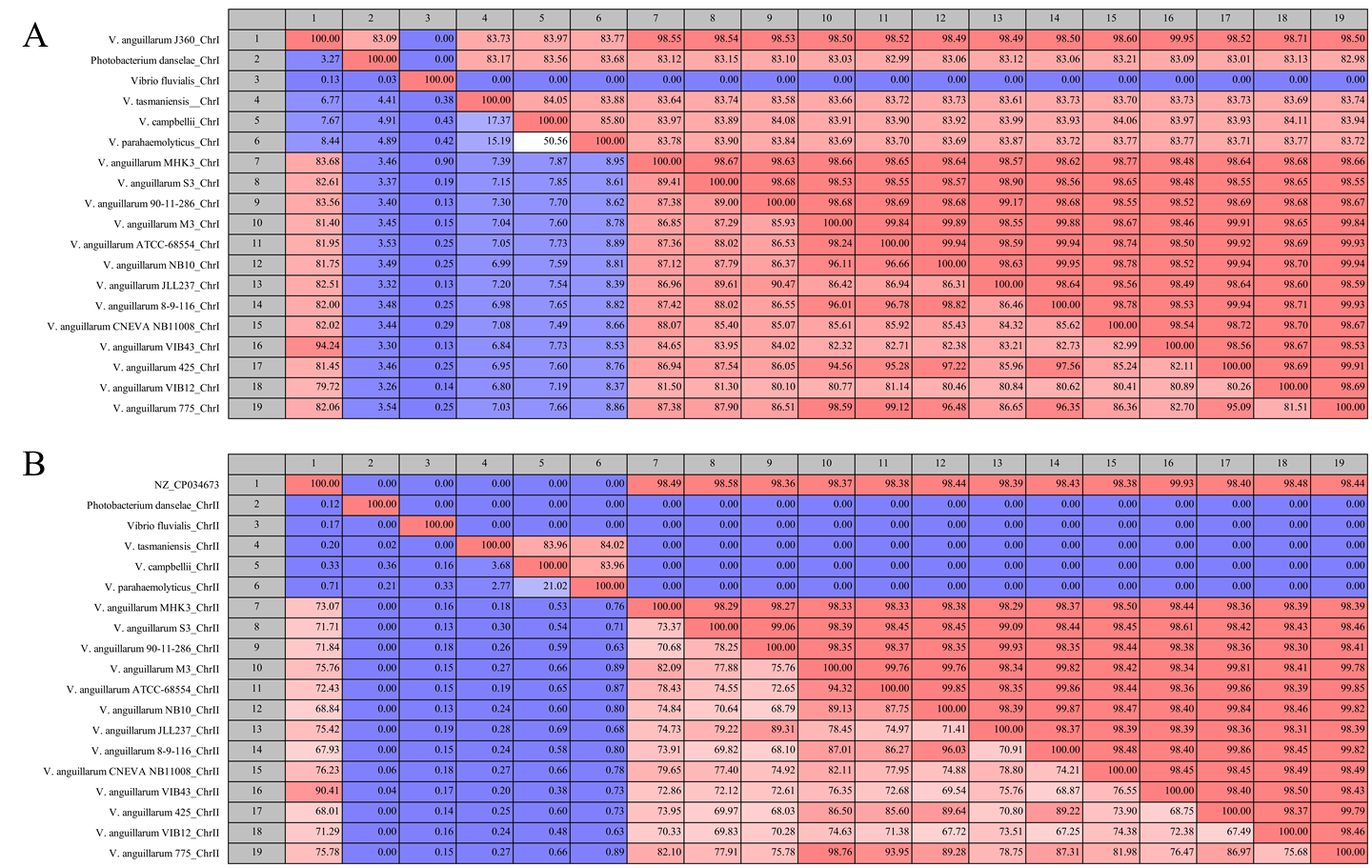
**

**Figure S2. Average nucleotide identity (ANI) comparison of *V. anguillarum* whole genome alignment. A.** *V. anguillarum* chromosome-I; **B.** *V. anguillarum* chromosome-II. Identity percentage parameters for annotated genes were set up as minimum similarity of 0.8 and minimum length 0.8. Analyses were conducted in CLC Genomic Workbench v20 (CLC Bio). ANIs tables indicate that *V. anguillarum* J360 and *V. anguillarum* VIB43 possess about 99.95% of identity within chromosome-I and 99.93% within chromosome -II.

**
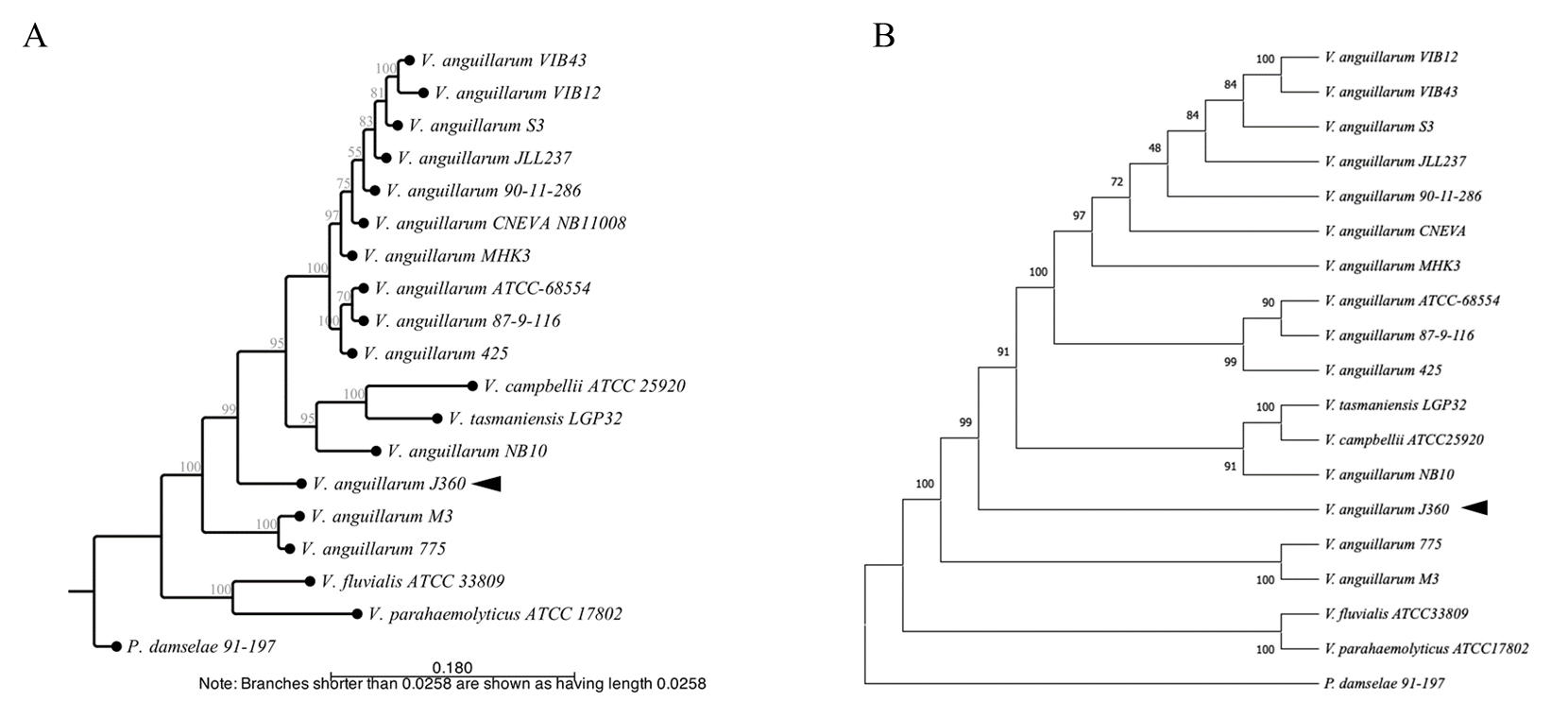
**

**Figure S3. Phylogenetic analysis of *V. anguillarum* using Multi Locus Sequence Analysis (MLA). A.** Phylogenetic analysis conducted in CLC Workbench v20 (CLC Bio). **B.** Phylogenetic analysis conducted in MEGA X. The evolutionary distance was calculated using the Neighbor-Joining method with a bootstrap consensus from 500 replicates. Evolutionary distance was computed using Jukes-Cantor method. All ambiguous positions were removed for each sequence pair (pairwise deletion option). The analysis involved 9 loci (16S rRNA, *fstZ*, *gapA*, *gyrB*, *mreB*, *pyrH*, *recA*, *rpoA*, *topA*) from 18 complete genomes of *Vibrio* species and *Ph. damselae* 91-197 as an outgroup. Phylogenetic analysis was conducted using CLC NGS workbench v20 (CLC Bio).


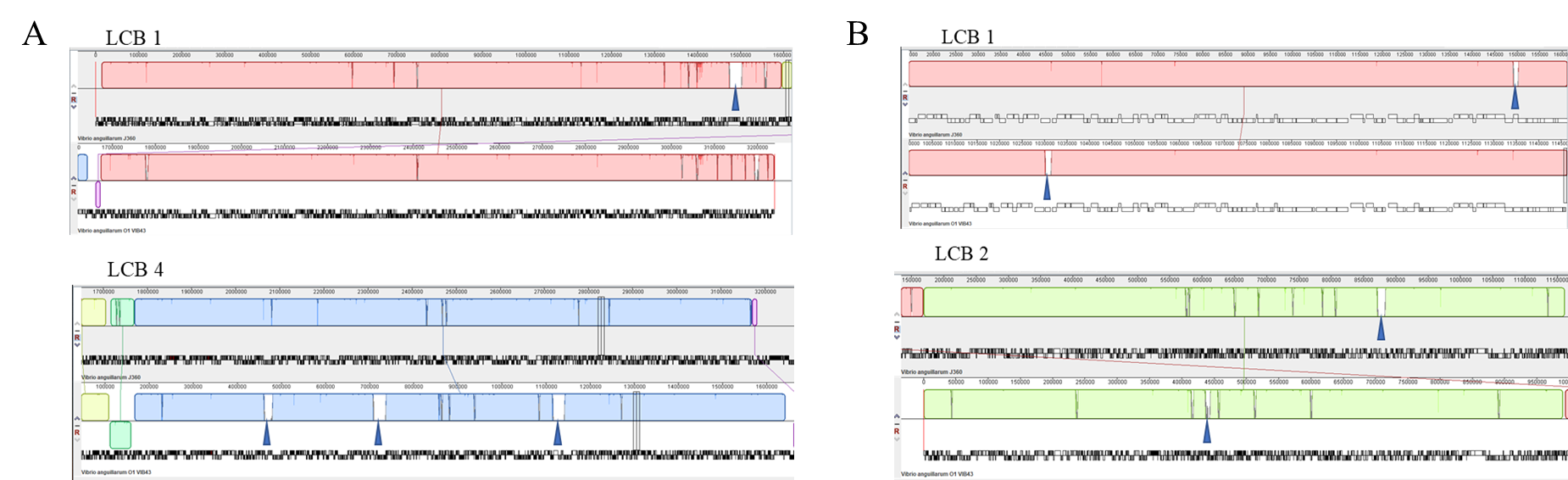


**Figure S4. Comparative whole genome alignment closeup between *V. anguillarum* J360 and *V. anguillarum* VIB43. A.** LCBs-1 and -3 of chromosome-I. **B.** LCBs-1 and -2 of chromosome-II. Solid Arrows represent genome gaps (GGs).


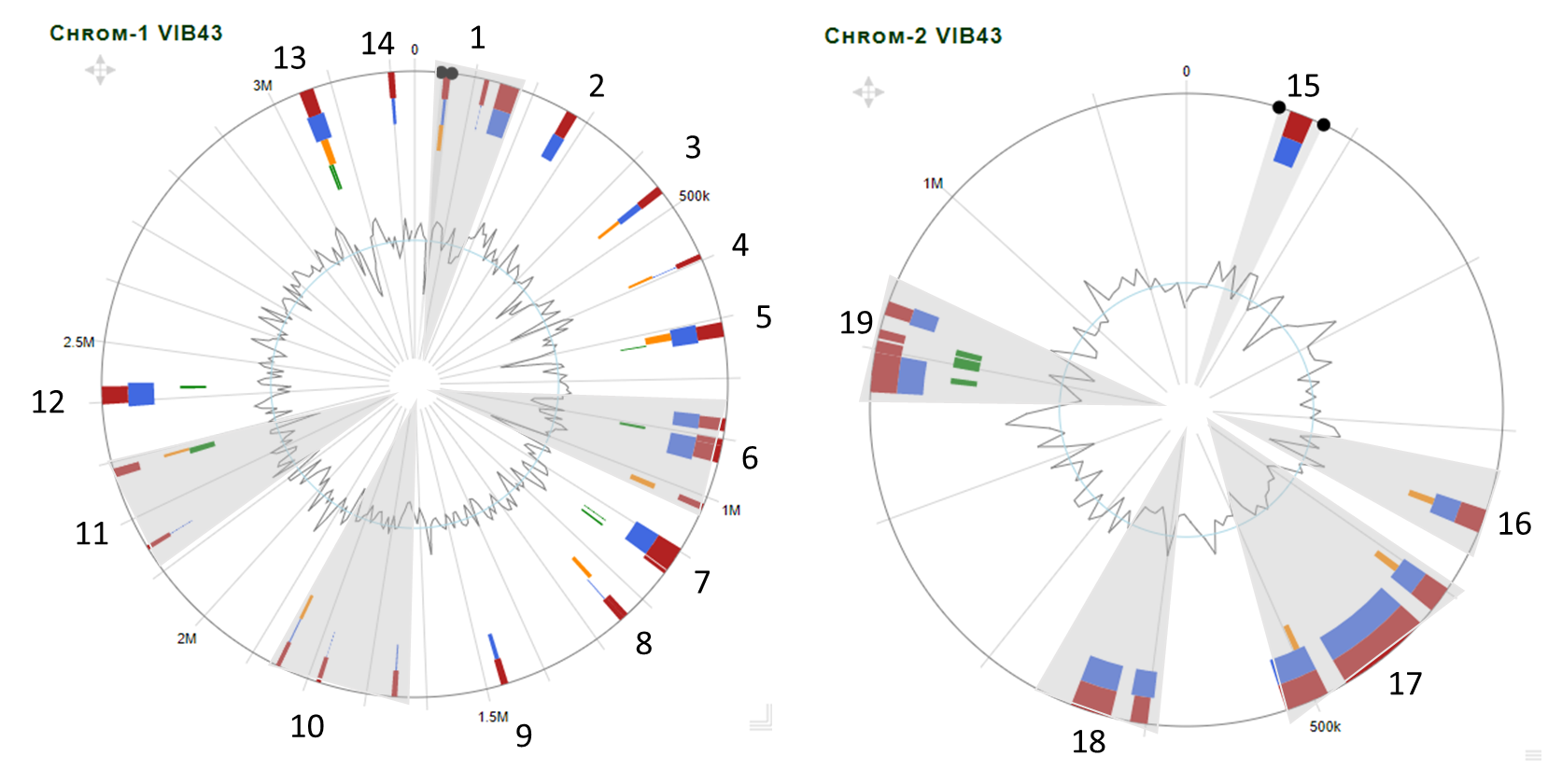


**Figure S5. Comparative analysis *V. anguillarum* J360 large plasmid pVaJ360-I. A.** Heat map visualization of aligned sequences identity for large plasmid pVaJ360-I. **B.** Average nucleotide identity (ANI) comparison of pVaJ360-I. Identity percentage parameters for annotated genes were set up as minimum similarity of 0.8 and minimum length 0.8. Analyses were conducted in CLC Genomic Workbench v20 (CLC Bio). The analysis involved five *V. anguillarum* large plasmids: pJM1 (AY312585), p67 (LK021128), p65 (CP023210), p15 (CP0230,56) and pVaJ360. The analysis was conducted using CLC Workbench v20 (CLC Bio). ANI table and heat map indicate that large plasmid pVaJ360 does not possess identity within pJM1 or pJM1-like virulent plasmids.

**
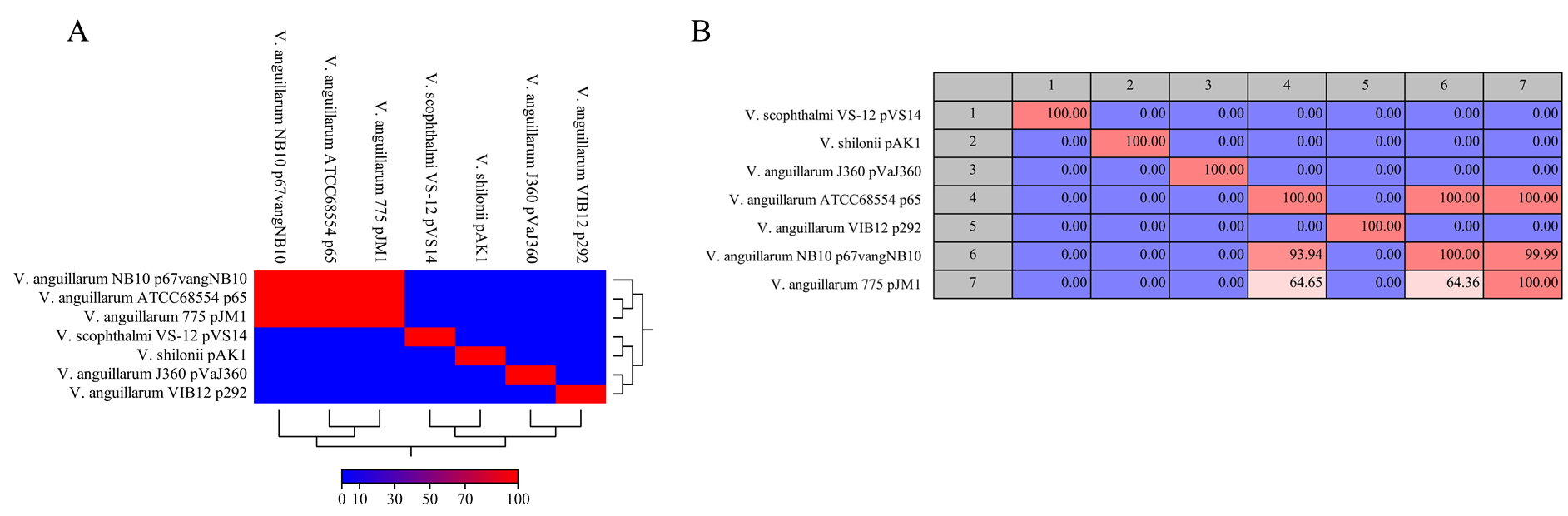
**

**Figure S6. *V. anguillarum* VIB43 genomic islands (GIs). A.** Genomic islands (GIs) detected in chromosome-I; Genomic islands (GIs) detected in chromosome-II. Red bars represent GIs detected using 3 different packages; blue bars represent GIs detected with SIGI-HMM package; orange bars represent GIs detected with IslandPath-DIMOB package; green bars represent the GIs detected with IslandPick package.
