# Supplementary material for: Comparative Genomics Analysis of Vibrio anguillarum Isolated from Lumpfish (Cyclopterus lumpus) in Newfoundland Reveal Novel Chromosomal Organizations
Source: Microorganisms. 2020 Oct 27;8(11):1666. doi: 10.3390/microorganisms8111666 (PMC7716436; doi:10.3390/microorganisms8111666)
Supplement: Supplementary file 1 [file microorganisms-08-01666-s001.zip › Supplementary/Supplementary Tables. docx.docx]

**Supplementary Data**

**Table S1.** Enzymatic profile of *V. anguillarum* J360 using the API ZYM system

| Enzyme assay for: | Reaction |
| --- | --- |
| Alkaline phosphatase | + |
| Esterase (C_4_) | + |
| Esterase lipase (C_8_) | + |
| Lipase (C_14_) | + |
| Leucine arylamidase | + |
| Valine arylamidase | + |
| Cystine arylamidase | + |
| Trypsin | - |
| α-Chymotrypsin | - |
| Acid phosphatase | + |
| Naphthol-AS-BI-Phosphohydrolase | - |
| α-Galactosidase | - |
| β-Galactosidase | - |
| β-Glucoronidase | - |
| α-Glucosidase | - |
| N-Acetyl-β-glucosaminidase | - |
| α-Mannosidase | - |
| α-Fucosidase | - |

**Table S2.** Enzymatic profile of *V. anguillarum* J360 using API20NE

| Enzyme assay for | Reaction |
| --- | --- |
| Reduction of nitrates to nitrites | + |
| Indole production | + |
| Glucose fermentation | + |
| Urease | + |
| β-Galactosidase | + |
| Hydrolysis of: |  |
| Arginine | + |
| Esculin | + |
| Gelatin | + |
| Assimilation of: |  |
| D-Glucose | + |
| L-Arabinose | + |
| D-Mannose | + |
| D-Mannitol | + |
| N-acetyl-glucosamine | + |
| D-maltose | + |
| Potassium gluconate | + |
| Capric acid | - |
| Adipic acid | - |
| Malic acid | + |
| Trisodium citrate | + |
| Phenylacetic acid | - |
| Code | 7777745 |
| Result | Possible *V. fluvialis* |

**Table S3.** Enzymatic profile of *V. anguillarum* J360 using API20E

| Enzyme Assayed for | Reaction |
| --- | --- |
| β-Galactosidase | + |
| Indole production | - |
| Acetoin production | + |
| Citrate utilization | + |
| H_2_S production | - |
| Urease | + |
| Hydrolysis of: |  |
| L-Arginine | + |
| L-Lysine | + |
| L-Ornithine | - |
| L-Tryptophane | - |
| Gelatinase | + |
| Assimilation of: |  |
| D-Glucose | + |
| D-Mannitol | - |
| Inositol | - |
| D-Sorbitol | - |
| L-Rhamnose | - |
| D-Saccharose | - |
| D-Melibiose | - |
| D-Amygdaline | - |
| L-Arabinose | - |
|  |  |

**Table S4.** Housekeeping genes used for MLSA.

| **Housekeeping gene and**  **size (bp)** | **Description** | ***V. anguillarum* gene locus tags** |
| --- | --- | --- |
| 16S rRNA (1,544 bp) | ribosomal RNA subunit | VAA_r008; VANGB10 (233624..235167); N175_01225; CMV05_00360; CK207_00110; CEQ50_00475; CG015_01435; B5S57_08110; CEA93_00375; CEJ46_00360; CLI14_00110; PL14_00290; CEG15_00290; A8140_00610; AL536_07795; AL464_04065; VS_r0045 |
| *ftsZ* (1,148 bp) | cell-division protein | VAA_RS03925; VANGNB10_cI2165c; N175_04065; CMV05_12545; CK207_12375; CEQ50_11740; CG015_11710; B5S57_08595; CEA93_11805; CEJ46_11435; CLI14_02890; PL14_10850; CEG15_10675; A8140_02565; AL536_17830; AL464_01670; VS_0454 |
| *gapA* (1,443 bp) | glyceraldehyde-3-phosphate dehydrogenase | VAA_RS09935; VANGNB10_cI0936c; N175_15935; CMV05_17185; CK207_05935; CEQ50_05455; CG015_05975; B5S57_15270; CEA93_05315; CEJ46_05345; CLI14_09005; PL14_04780; CEG15_04820; A8140_10660; AL536_01340; AL464_04430; VS_0932 |
| *gyrB* (2,418 bp) | gyrase beta subunit | VAA_RS01005; VANGNB10_cI0013; N175_01055; CMV05_00065; CK207_15105; CEQ50_00155; CG015_01215; B5S57_05625; CEA93_00065; CEJ46_00065; CLI14_14270; PL14_00020; CEG15_00065; A8140_00300; AL536_11690; AL464_14755; VS_0013 |
| *mreB* (1,044 bp) | rod shape-determining protein | VAA_RS13485; VANGNB10_cI0292; N175_13945; CMV05_01690; CK207_01990; CEQ50_02110; CG015_02870; B5S57_18600; CEA93_01790; CEJ46_01640; CLI14_12555; PL14_01680; CEG15_01650; A8140_13375; AL536_18720; AL464_05080; VS_0341 |
| *pyrH* (732 bp) | uridine monophosphate (UMP) kinase or uridylate kinase | VAA_RS10055; VANGNB10_cI2045c; N175_04705; CMV05_11900; CK207_11775; CEQ50_11105; CG015_11100; B5S57_09200; CEA93_11190; CEJ46_10830; CLI14_03495; PL14_10250; CEG15_10075; A8140_11485; AL536_09140; AL464_07985; VS_2351 |
| *recA* (1,047 bp) | recombinase A | VAA_RS12550; VANGNB10_cI0448; N175_12945; CMV05_02370; CK207_02730; CEQ50_02830; CG015_03445; B5S57_17775; CEA93_02675; CEJ46_02620; CLI14_11580; PL14_02240; CEG15_02305; A8140_10030; AL536_18120; AL464_06820; VS_2596 |
| *rpoA* (993 bp) | RNA polymerase alpha subunit | VAA_RS03025; VANGNB10_cI2326c; N175_03130; CMV05_13510; CK207_13280; CEQ50_12630; CG015_12595; B5S57_07715; CEA93_12685; CEJ46_12320; CLI14_01985; PL14_11715; CEG15_11560; A8140_01855; AL536_22115; AL464_02385; VS_2807 |
| *topA* (2,631 bp) | DNA topoisomerase I | VAA_RS09170; VANGNB10_cI1085; N175_09445; CMV05_00315; CK207_00385; CEQ50_06215; CG015_06725; B5S57_14500; CEA93_06085; CEJ46_06100; CLI14_08235; PL14_05545; CEG15_05570; A8140_00570; AL536_12085; AL464_14115; VS_1081 |
